# Horizontal Gene Transfer From Bacteria and Plants to the Arbuscular Mycorrhizal Fungus *Rhizophagus irregularis*

**DOI:** 10.3389/fpls.2018.00701

**Published:** 2018-05-25

**Authors:** Meng Li, Jinjie Zhao, Nianwu Tang, Hang Sun, Jinling Huang

**Affiliations:** ^1^Key Laboratory for Plant Diversity and Biogeography of East Asia, Kunming Institute of Botany, Chinese Academy of Sciences, Kunming, China; ^2^University of Chinese Academy of Sciences, Beijing, China; ^3^Department of Economic Plants and Biotechnology, Yunnan Key Laboratory for Wild Plant Resources, Kunming Institute of Botany, Chinese Academy of Sciences, Kunming, China; ^4^Institute of Plant Stress Biology, State Key Laboratory of Cotton Biology, Henan University, Kaifeng, China; ^5^Department of Biology, East Carolina University, Greenville, NC, United States

**Keywords:** arbuscular mycorrhizal fungi, endobacteria, eukaryotic evolution, horizontal gene transfer, symbiosis

## Abstract

Arbuscular mycorrhizal fungi (AMF) belong to Glomeromycotina, and are mutualistic symbionts of many land plants. Associated bacteria accompany AMF during their lifecycle to establish a robust tripartite association consisting of fungi, plants and bacteria. Physical association among this trinity provides possibilities for the exchange of genetic materials. However, very few horizontal gene transfer (HGT) from bacteria or plants to AMF has been reported yet. In this study, we complement existing algorithms by developing a new pipeline, Blast2hgt, to efficiently screen for putative horizontally derived genes from a whole genome. Genome analyses of the glomeromycete *Rhizophagus irregularis* identified 19 fungal genes that had been transferred between fungi and bacteria/plants, of which seven were obtained from bacteria. Another 18 *R. irregularis* genes were found to be recently acquired from either plants or bacteria. In the *R. irregularis* genome, gene duplication has contributed to the expansion of three foreign genes. Importantly, more than half of the *R. irregularis* foreign genes were expressed in various transcriptomic experiments, suggesting that these genes are functional in *R. irregularis*. Functional annotation and available evidence showed that these acquired genes may participate in diverse but fundamental biological processes such as regulation of gene expression, mitosis and signal transduction. Our study suggests that horizontal gene influx through endosymbiosis is a source of new functions for *R. irregularis*, and HGT might have played a role in the evolution and symbiotic adaptation of this arbuscular mycorrhizal fungus.

## Introduction

Horizontal gene transfer (HGT) is the movement and integration of genetic material between phylogenetically distant or unrelated species. It is a well-known and pervasive evolutionary mechanism in prokaryotes (Gogarten, 2003; Soucy et al., 2015). Previous research revealed that an average of approximately 81% genes in 181 sequenced prokaryotic genomes have been subject to HGT in a long history of prokaryotic evolution (Dagan et al., 2008). In prokaryotic genomes, transferred genes are responsible for the majority of protein families' expansion (Treangen and Rocha, 2011). HGT events also occur in all major groups of eukaryotes though they were initially thought to be rare (Bock, 2010; Huang, 2013). For instance, studies based on available genomic data have uncovered that 57 gene families in the moss *Physcomitrella patens* genome were obtained from prokaryotes, fungi or viruses (Yue et al., 2012). Transcriptomic analyses of the bdelloids rotifers indicated that about 10% of active genes had a foreign origin (Boschetti et al., 2012). In fungi, 713 transferred genes were detected in 60 sequenced genomes (Marcet-Houben and Gabaldon, 2010). One of the exemplary systems to understand HGT in eukaryotes is parasitic plants, in which high rates of HGT were observed (e.g., Orobanchaceae) (Davis and Xi, 2015; Yang et al., 2016).

The integration and fixation of horizontally transferred genes can benefit the recipient organism by introducing a new function (Koonin et al., 2001; Lacroix and Citovsky, 2016). HGT could improve the adaptive ability of prokaryotes in changing environments (Popa and Dagan, 2011), as evidenced by the role of HGT in the spread of antibiotic resistance in bacterial pathogens such as *Clostridium difficile* and *Staphylococcus aureus* (Juhas, 2015). In eukaryotes, HGT is less frequent than in prokaryotes but still important for adaptive evolution (Husnik and McCutcheon, 2017; McInerney, 2017). For instance, horizontally acquired genes in *P. patens* are involved in diverse biological processes (e.g., xylem formation, plant defense, hormone biosynthesis), which were thought to be important for the transition of green plants from aquatic to terrestrial environments (Yue et al., 2012). Other examples include bdelloid rotifers, in which the accumulation of deleterious mutations from asexual reproduction might be prevented by acquiring new genes to replace the defective ones (Boschetti et al., 2012), and plant-parasitic nematodes where HGTs have promoted parasitism (Danchin et al., 2010; Whiteman and Gloss, 2010; Mayer et al., 2011).

Arbuscular mycorrhizal fungi (AMF) belong to the mono-subphylum Glomeromycotina. About 80% of land plants can form mutualistic symbioses with AMF (Spatafora et al., 2016). In this symbiotic association, AMF help plants obtain nutrients such as nitrogen and phosphorus (Bonfante and Genre, 2010; Smith and Smith, 2011), and in return the plants supply the fungus with lipids and sugars (Pfeffer et al., 1999; Bago et al., 2002; Trépanier et al., 2005; Jiang et al., 2017; Wang et al., 2017). As obligate biotrophs inhabiting plant roots inter- and intra-cellularly, AMF also harbor diverse endobacteria in their cytoplasm (Naumann et al., 2010), in addition to the many other bacteria in the mycorrhizosphere (Bonfante and Anca, 2009). The intimate association between AMF and bacteria/plants thus provides opportunities for genetic material exchange between them (Skippington and Ragan, 2012). Indeed, recent surveys of Mollicutes-related endobacteria (MRE) genomes detected genes that might have been transferred from ancestral AMF (Naito et al., 2015; Torres-Cortes et al., 2015). However, till now very few HGTs from bacteria or plants to AMF have been reported (Lee et al., 2018). *Rhizophagus irregularis* was the first arbuscular mycorrhizal fungus whose genome has been sequenced (Tisserant et al., 2013; Lin et al., 2014), allowing us to comprehensively explore whether HGTs have occurred in AMF and, if so, how acquired genes affected the evolution and adaptation of AMF. In this study, we investigate the occurrence of HGTs in *R. irregularis* using our HGT detection pipeline. The potential roles of acquired genes in the adaptation and evolution of this organism are further discussed.

## Materials and methods

### Data sources

The *R. irregularis* genome, proteome and annotation files released by Tisserant et al. were downloaded from the Joint Genome Institute (JGI, http://genome.jgi.doe.gov/Gloin1/Gloin1.download.html) (Tisserant et al., 2013). Forty-one transcriptomes of *R. irregularis* were obtained from NCBI BioProject via the following accessions: PRJDB3195 and PRJNA287285. Draft genomes of four *R. irregularis* strains (A1, A4, A5, and C2) were downloaded from NCBI genome database (https://www.ncbi.nlm.nih.gov/genome/genomes/18237) and JGI website (https://genome.jgi.doe.gov/Rhiir2_1/Rhiir2_1.home.html) according to previous researches (Ropars et al., 2016; Chen et al., 2018).

### BLAST procedure

The annotated protein sequences of *R. irregularis* were used to search against the NCBI Reference Sequence Database (RefSeq release 81) and nonredundant protein sequence database (NR). For bacteria (taxonomy ID 2) and plants (taxonomy ID 33090), RefSeq and NR subset databases were additionally generated and searched. Outputs from above BLAST procedures were merged for following analyses.

### Screening of HGT candidates in *R. irregularis* genome

Two similar measurements are often used to screen for transferred gene: HGT index (*h*) (Boschetti et al., 2012) and alien index (AI) (Gladyshev et al., 2008), both of which depend on BLAST results. Here HGT index is defined as the bit score difference between the best match from a donor group and that from a closely related group (Boschetti et al., 2012; Crisp et al., 2015). Alien index is calculated by the formula AI = log[(best *E*-value for donors) + 1e^−200^]–log[(best *E*-value for non-donors) + 1e^−200^] (Gladyshev et al., 2008). HGT index has been recently reported to be a successor to alien index because it would not be affected by the size of BLAST database (Crisp et al., 2015).

To rapidly and efficiently calculate *h* and AI, we developed a HGT detection pipeline: Blast2hgt. Blast2hgt reads in BLAST results and parses best *E*-value (i.e., minimum *E*-value) and bit score (i.e., maximum bit score) for each taxonomy group. AI and *h* are calculated accordingly and then used to evaluate whether HGT events have occurred and the likelihood of transfers. In this study, bacteria and plants were considered to be potential donor groups. A gene was considered to be a viable HGT candidate when both HGT index and alien index led to the same conclusion of possible HGT. Blast2hgt uses MySQL to speed up querying the organism information (e.g., *Arabidopsis thaliana*) and the taxonomic group (e.g., plants) of a given BLAST hit. More importantly, the cache mechanism of Blast2hgt can greatly facilitate the analysis when BLAST outputs are extremely large. In addition, because NCBI BLAST outputs a maximum of 500 hits by default (increasing max_target_seqs costs huge memory, which is unbearable for most desktop PCs), some taxonomic groups may not be covered in BLAST results. One of the strategies is to split the BLAST database into smaller ones according to taxonomic groups and then BLAST against them separately. Blast2hgt also implements a script to carry out this procedure. This pipeline is open source and available at GitHub (https://github.com/waterml/blast2hgt).

### Conserved domain and gene family prediction

Conserved domains were identified using HMMER searches against the PFAM database 27 (Eddy, 2011; Finn et al., 2016), and online searches against the NCBI conserved domain database (CDD) (Marchler-Bauer et al., 2017) and SMART database (Simple Modular Architecture Research Tool) (http://smart.embl.de/). Orthologs in the putative horizontally derived genes were identified using OrthoMCL (Li et al., 2003). Gene families were predicted from the OrthoMCL results if domains were shared between sequences.

### Contamination elimination

Possible contamination was eliminated by manually inspecting whether genes on the same genomic scaffold had close relationships with putative donor groups. The scaffolds used here included those from initial genome assembly (Tisserant et al., 2013) and genomes of four other strains (A1, A4, A5, and C2) (Ropars et al., 2016; Chen et al., 2018). If all neighboring genes on the scaffold were more similar to sequences from a foreign group (e.g., bacteria or plants) than those from more closely related taxa, that scaffold would be treated as contamination. Furthermore, to ensure that HGT gene is not contamination in bacteria or plants, the presence of gene homologs in at least five bacterial species (or plants if the transfer occurred between *Rhizophagus*/fungi and plants) was required.

### Determination of transferred genes

Taxonomic distributions and phylogenetic relationships were examined to validate the transferred genes. For the taxonomic distribution method, two requirements were imposed: (1). the transferred gene should not have BLAST hits in any other taxonomic groups outside the putative donor and recipient (*E*-value cutoff of 1e-3); (2). BLAST hits in putative donor and recipient groups (*E*-value cutoff of 1e-5) should share conserved domains or similar sites. For HGT candidates not meeting above criteria or with undetermined transfer direction, phylogenetic analyses would be performed. Protein sequences from representative taxonomic groups of three domains of life (bacteria, archaebacteria and eukaryotes; see Supplementary Methods for details) were sampled from RefSeq and NR, and the constructed phylogenies based on the two databases were compared. During the sampling process, manual inspection and additional samplings would be performed whenever necessary. Multiple sequence alignments were performed using MAFFT (v7.205) (Katoh and Standley, 2013). Poorly aligned regions and gaps were removed using trimAl (v1.4) (Capella-Gutierrez et al., 2009). Maximum likelihood phylogenetic trees were reconstructed using IQ-TREE (v1.5.4) (Nguyen et al., 2015) with automatically selected best-fit amino acid substitution model. Branch supports were estimated by 1000 ultrafast bootstrap and SH-like approximate likelihood ratio test implemented in IQ-TREE. Bayesian analyses were conducted with MrBayes 3.2.6 (Ronquist et al., 2012). Two independent runs with four chains each were calculated simultaneously for ten million generations, sampling every 100 generations. The average standard deviation of split frequencies below 0.01 was used to ensure convergence of the runs. The posterior probability values were generated after discarding the first 25% of the sampled trees. Phylogenetic trees were visualized using MEGA 7.0 (Kumar et al., 2016) and Figtree (v1.4.3, http://tree.bio.ed.ac.uk/software/figtree/). Because NR sampling was often found to contain contamination sequences, manual inspection was performed when HGT conclusion was affected by discrepancy between RefSeq sampling and NR sampling.

### Transfer direction

For horizontally transferred genes with a wide distribution in both donor and recipient lineages, as well as in other taxonomy groups, phylogenetic trees were reconstructed to investigate the transfer polarity. For genes only found in donor and recipient groups (i.e., see taxonomic distribution method above), a credible hypothesis assumes that the source of gene transfer should be the taxon that has the most diverse representation of the given gene family (Koonin et al., 2001). It is less likely for several organisms to obtain a gene independently from a single species at the same time.

### Transcriptomic analysis

RNA-seq data were evaluated using the FastQC software (http://www.bioinformatics.babraham.ac.uk/projects/fastqc/). Qualified reads were aligned using HISAT2 (v2.1.0) (Pertea et al., 2016). The expression level in each sample was quantified using StringTie (v1.3.3b) (Pertea et al., 2015, 2016).

## Results

### Analytical schema for determining HGTs

**Figure 1:**
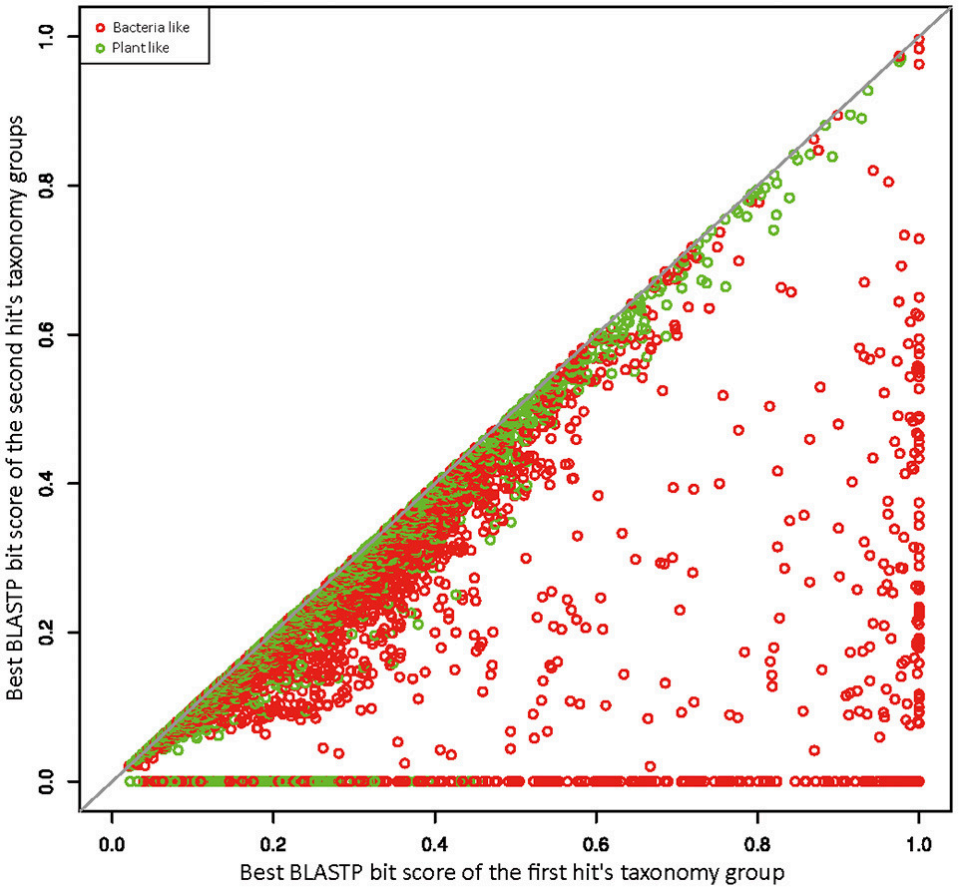
Similarity plot of *R. irregularis* bacteria- and plant-like proteins. Circles represent relative bit scores of *R. irregularis* proteins. The x axis is the best bit score produced by BLASTP against bacteria/plants (first hit's taxonomy group). The y axis is the best bit score generated by BLASTP against second hit's taxonomy group (which contains all sequences not belonging to first hit's taxonomy group and fungi). To get the relative bit score, actual bit score is divided by bit score of the query sequence BLASTP against itself.

To better explore the relationships between *R. irregularis* proteins and their BLAST hits, we employed a scatter plot to visualize sequence similarities (Maumus et al., 2014). For each query, the BLASTP bit score was recorded from its best match. After excluding all hits from the first hit's taxonomy group, a second BLASTP score was obtained from the best match of remaining hits. In this study, the first hit's taxonomy group could be green plants (taxonomy ID 33090) or bacteria (taxonomy ID 2), and the second hit's taxonomy groups contain all hits that did not belong to the first hit's taxonomy group or fungi. The first and second BLASTP scores were both normalized by dividing the bit score generated by query sequence against itself. These normalized scores from the first and second hit's taxonomy groups were plotted against each other along x and y axes. If x is greater than the corresponding y, the focal gene will be more similar to sequences from distantly related taxa (i.e., green plants or bacteria) than those from close relatives, which would suggest a potential HGT event. 4126 genes have their best match in bacteria or plants (Figure 1), suggesting signals of relatedness to the two groups. However, these signals could be derived from contamination in genome sequencing samples because of the overall poor quality of *R. irregularis* genome assembly (scaffold N50: 5997 bp). Hence, to eliminate false positives in HGT detection, we manually inspected genes neighboring to HGT candidates on the same scaffold. If one or more neighboring genes belonged to native fungal genes, the scaffold was deemed not to be a contamination. A HGT candidate would be removed from further consideration if it was located on a short scaffold with no flanking genes. Phylogenetic analyses were performed on HGT candidates that have identifiable homologs in multiple major taxonomic groups (i.e., sequences not restricted to fungi, bacteria and/or plants). The detailed analysis flowchart is shown in Supplementary Figure 1.

### Bacteria- and plant-like genes in *R. irregularis*

**Figure 2:**
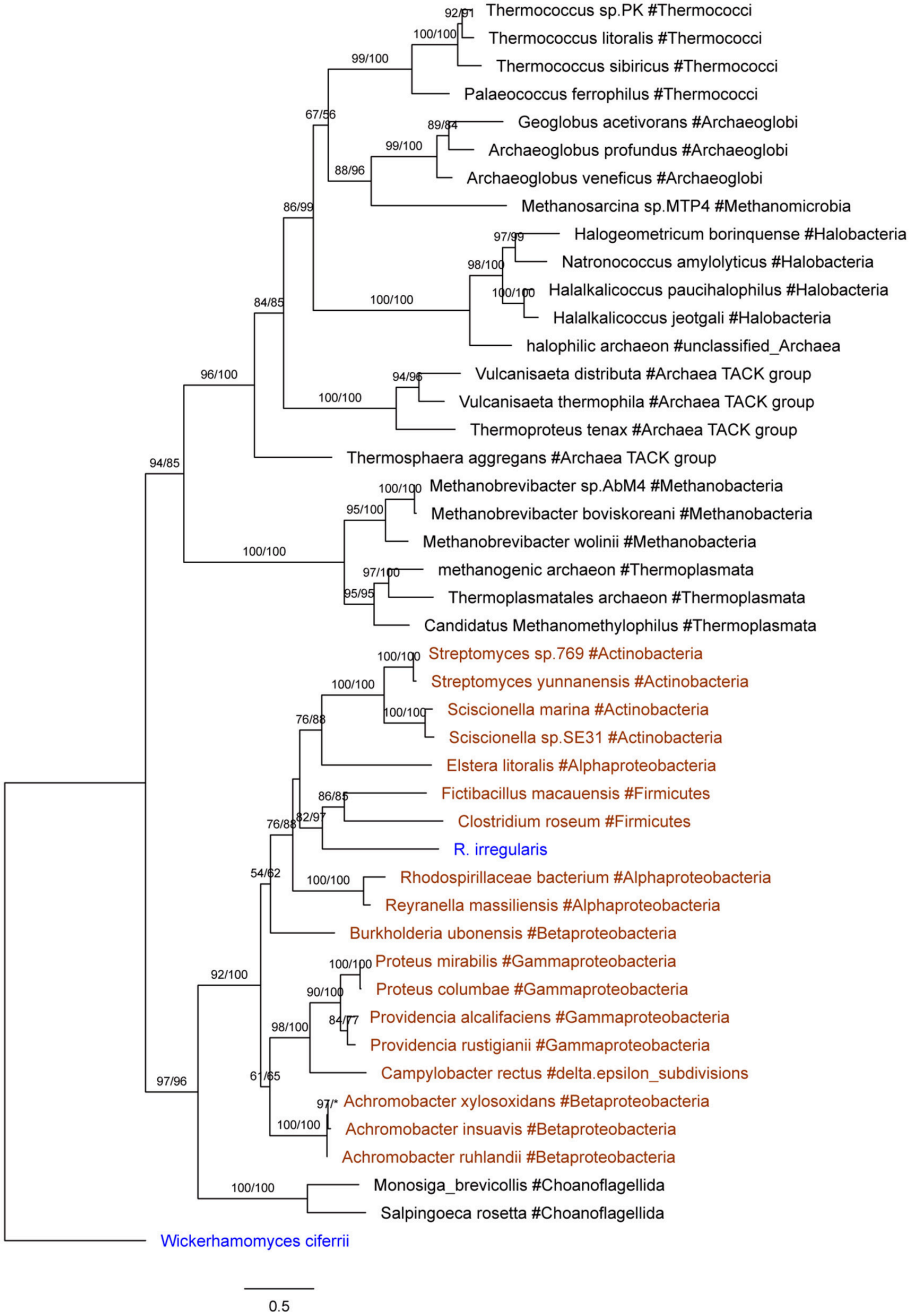
Molecular phylogeny of YbaK/prolyl-tRNA synthetases. Protein sequence of the fungus *Wickerhamomyces ciferrii* (XP_011275603.1) is used as the outgroup. Numbers beside branches represent bootstrap values from maximum likelihood and Bayesian analyses, respectively. Asterisks indicate values lower than 50%. Scale bars represent substitution numbers per amino-acid site. Bacterial and fungal sequences are colored in orange and blue, respectively.

Seven *R. irregularis* genes, including genes encoding an ATPase (ESA12330.1), a nucleotidyltransferase (ESA03915.1), a cytotoxin (ESA20238.1) and four hypothetical proteins (ESA06580.1, ESA03029.1, ESA22708.1, and ESA22410.1), had more than one homolog in different groups of bacteria (*E*-value cutoff: 1e-6), but none in any other fungi (*E*-value cutoff: 1e-3). HGT and gene loss are the most probable explanations for such observations (Crisp et al., 2015). Given that fungi and bacteria are only distantly related to each other, differential gene loss is a less parsimonious explanation, since it would need numerous losses in different groups (Soucy et al., 2015). Hence, these genes were considered as horizontally transferred genes from bacteria to *R. irregularis*. It is possible that these genes might also be found in other *Rhizophagus* when their genome data become available. In this study, these genes specifically acquired by *R. irregularis* or its close relatives are defined as recent transfers. In addition, phylogenetic analyses suggest that four *R. irregularis* genes, including a YbaK/prolyl-tRNA synthetase, a class I SAM-dependent methyltransferase, a polo kinase and a gene of unknown function, were likely obtained from bacteria (Figure 2 and Supplementary Figures 2–4). All these genes are closely related to bacterial sequences, but they are either distantly related to or have no detectable fungal homologs in our analyses, suggesting recent HGTs from bacteria to *R. irregularis* or its close relatives. An example for such a relationship is the gene encoding YbaK/prolyl-tRNA synthetase. Phylogenetic analyses clearly show that this *R. irregularis* sequence is derived from bacteria (Figure 2).

Similarly, five *R. irregularis* genes had homologs in multiple major plant groups (e.g., angiosperms, bryophytes, or green algae) (*E*-value cutoff: 1e-6), but not in other fungi (*E*-value cutoff: 1e-3). These genes encoded a transposase family (ESA21548.1), a pentatricopeptide repeat (PPR) protein (ESA06270.1), a HAUS augmin-like protein (ESA01078.1), a retrotransposon protein (ESA16847.1) and a hypothetical protein (ESA06626.1). Each of these genes (and the transposase gene family) is present in diverse plant lineages, but absent from other fungi, suggesting that they were likely recently transferred from plants to *R. irregularis* (Table 1). Two other *Rhizophagus* genes (i.e., genes encoding a calcium binding protein and a protein kinase) also appear to be acquired from plants according to the phylogenetic analyses (Supplementary Figures 5, 6).

**Table 1:** Genes acquired by *R. irregularis* and other fungi.

**Protein ID^a^**	**NCBI accession**	**Donor**	**Best hit in donor**	**Putative function**	**Conserved domains**
17338^b^	ESA21548.1	Plants	XP_015965100.1	Hypothetical protein	MULE transposase
33937^b^	ESA06626.1	Plants	XP_002952900.1	Hypothetical protein	NA
349874^b^	ESA06270.1	Plants	XP_019104751.1	Pentatricopeptide repeat protein	PPR
22379^b^	ESA16847.1	Plants	XP_015624409.1	Retrotransposon protein	NA
338156^b^	ESA01078.1	Plants	XP_006430957.1	HAUS augmin-like complex subunit 5	HAUS5
9951	ERZ99026.1	Plants	XP_001698359.1	Protein kinase	Protein kinase
349121	ESA08389.1	Plants	XP_013906219.1	Calcium binding protein	Caleosin
34003^b^	ESA06580.1	Bacteria	WP_080043528.1	Hypothetical protein	NA
184991^b^	ESA12330.1	Bacteria	WP_077765459.1	ATPase	DUF4325
334033^b^	ESA03915.1	Bacteria	WP_007910780.1	Nucleotidyltransferase	NA
636^b^	ESA03029.1	Bacteria	WP_027191624.1	Hypothetical protein	NA
91289^b^	ESA22708.1	Bacteria	WP_072830607.1	Hypothetical protein	NTP_transf_2
342680^b^	ESA22410.1	Bacteria	WP_047230252.1	Hypothetical protein	NYN
320082^b^	ESA20238.1	Bacteria	WP_046647651.1	Cytotoxin	Cytotoxic
1695	ESA22999.1	Bacteria	WP_070119383.1	Class I SAM-dependent methyltransferase	Methyltransf_11, SmtA
321933	ESA16208.1	Bacteria	WP_051718259.1	Hypothetical protein	NA
189658	ESA11624.1	Bacteria	WP_002170799.1	Polo kinase	ALP_like, PKc_like
346561	ESA14342.1	Bacteria	WP_080607998.1	YbaK/prolyl-tRNA synthetase	YbaK_like family
202831^c^	ESA09649.1	Bacteria	WP_009106322.1	Succinyl-CoA synthetase	DUF418
331093^c^	ERZ95723.1	Bacteria	XP_006679999.1	Ribokinase	PfkB
343228^c^	ESA21322.1	Bacteria	WP_034090265.1	Phosphoglycerate mutase	His_Phos_1
343557^c^	ESA20644.1	Bacteria	WP_020470151.1	Ketol-acid reductoisomerase	IlvN
343983^c^	ESA19705.1	Bacteria	WP_074926822.1	Chromate transporter	Chromate_transp
345690^c^	ESA16252.1	Bacteria	WP_052881627.1	Prephenate dehydratase	CM_2, PDT, ACT
337311^c^	ESA02570.1	Bacteria	WP_082348788.1	RNase III	RNaseIII

a *Protein IDs assigned by Tisserant et al. (2013)*.

b *Identified based on taxonomic distribution*.

c *Genes found in other fungi in addition to R. irregularis*.

Additionally, phylogenetic analyses also suggest that fungal progenitors (e.g., ancestors of Ascomycota, Basidiomycota and Chytridiomycota) gained genes from bacteria. Those genes encode a succinyl-CoA synthetase (ESA09649.1), a ribokinase (ERZ95723.1), a phosphoglycerate mutase (ESA21322.1), a RNase III (ESA02570.1), a ketol-acid reductoisomerase (ESA20644.1), a chromate transporter (ESA19705.1) and a prephenate dehydratase (ESA16252.1) (Supplementary Figures 7–13).

### Horizontally transferred genes from fungi to plants and between fungi and bacteria

**Figure 3:**
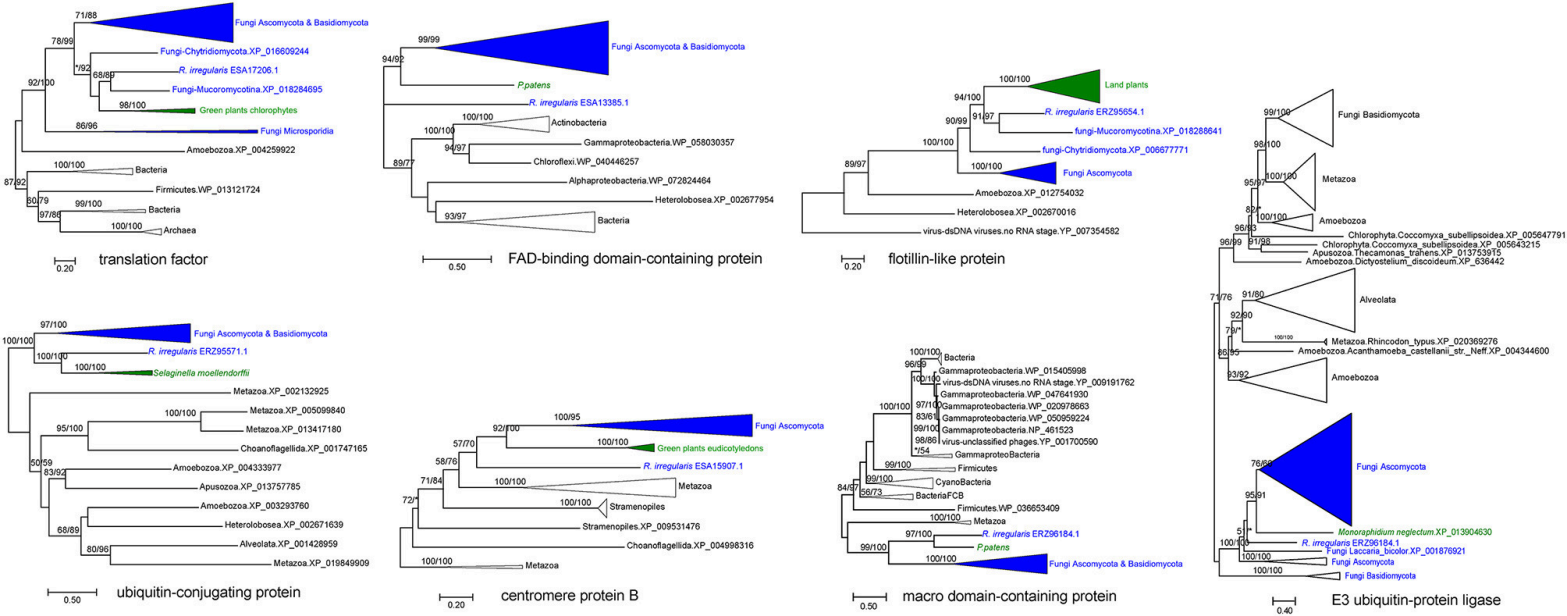
Molecular phylogenies of genes horizontally transferred between plants and fungi (including *R. irregularis*). Fungal and plant sequences were shown in blue and green, respectively. Subtrees containing sequences from the same taxonomic clade were condensed. Numbers beside branches represent bootstrap values from maximum likelihood and Bayesian analyses, respectively. Asterisks indicate values lower than 50%. Scale bars represent the number of amino acid substitutions per site. Detailed molecular phylogenies for these genes were displayed in Supplementary Figures 14–20.

Our analyses indicated that seven fungal genes were transferred to plants (Table 2). All of them were supported by molecular phylogenetic trees (Figure 3 and Supplementary Figures 14–20). For example, the molecular phylogeny of flotillin-like protein showed that the land plant sequence clade (including sequences from bryophytes, liverworts and angiosperms) was embedded within a paraphyletic group of fungal sequences (Figure 3 and Supplementary Figure 16), suggesting that the common ancestor of land plants acquired this gene from fungi. This observation is in agreement with the fact that basal land plants had established symbioses with Glomeromycota and Mucoromycotina (Ligrone et al., 2007; Field et al., 2015). Similar relationships were found for six other genes, which encode an E3 ubiquitin-protein ligase, a centromere protein B, a ubiquitin-conjugating protein, a macro domain-containing protein, a translation factor and a FAD-binding domain-containing protein (Figure 3 and Supplementary Figures 14–20). Notably, two moss *P. patens* genes encoding FAD-binding domain-containing protein (XP_001770677.1) and macro domain-containing protein (XP_001758106.1) were acquired from fungi. Both of them had no conserved homologs in any other plants, implying recent horizontal transfers. Similarly, the lycophyte *Selaginella moellendorffii* acquired a ubiquitin-conjugating protein encoding gene from fungi, and the chlorophyte *Monoraphidium neglectum* obtained an E3 ubiquitin-protein ligase gene of fungal origin.

**Table 2:** Fungi-derived genes in plants and genes horizontally transferred between fungi and bacteria (with undetermined directions).

**Protein ID^a^**	**NCBI accession**	**Homologous species**	**Hit in homologous species**	**Putative function**	**Conserved domains**
17795	ESA21136.1	*M. neglectum* (recipient)	XP_013904630.1	E3 ubiquitin-protein ligase	IBR
2547	ESA15907.1	Plants (recipient)	XP_010419288.1	Centromere protein B	HTH_Tnp_Tc5, DDE_1
331143	ERZ95654.1	Plants (recipient)	XP_011082203.2	Flotillin-like protein	Band_7
331170	ERZ95571.1	*S. moellendorffii* (recipient)	XP_002963132.1	Ubiquitin-conjugating protein	UQ_con
26097	ESA13385.1	*P. patens* (recipient)	XP_001770677.1	FAD-binding domain-containing protein	FAD_binding_4, BBE
340789	ERZ96184.1	*P. patens* (recipient)	XP_001758106.1	Macro domain-containing protein	Macro_2
40173	ESA17206.1	Plants (recipient)	XP_002947056.1	Translation factor	Sua5_yciO_yrdC
16636^b^	ESA22231.1	Bacteria (unknown direction)	WP_043735514.1	Unknown	NA
23706^b^	ESA15613.1	Bacteria (unknown direction)	WP_052890211.1	Methyltransferase	Methyltransf_25
24590^b^	ESA14777.1	Bacteria (unknown direction)	WP_013612302.1	Unknown	NA
28539^b^	ESA11217.1	Bacteria (unknown direction)	WP_045363948.1	Nacht nucleoside triphosphatase	NA
323897^b^	ESA11210.1	Bacteria (unknown direction)	WP_056049979.1	Phosphatidylserine decarboxylase	NA

a *Protein IDs assigned by Tisserant et al. (2013)*.

b *Identified based on taxonomic distribution*.

Five fungal genes (i.e., ESA22231.1, ESA15613.1, ESA14777.1, ESA11217.1, and ESA11210.1) have their closest homologs in bacteria (*E*-value cutoff: 1e-26) (Table 2). For these genes, no detectable homologs were found in other taxonomic groups outside bacteria and fungi (*E*-value cutoff: 1e-3), indicating that they were horizontally transferred between fungi and bacteria. However, the transfer directions for these five genes could not be predicted since they were distributed in more than one groups in both fungi and bacteria, and their molecular phylogenies failed to determine the transfer polarity clearly (Supplementary Figures 21–25).

### Expansion of acquired genes in *Rhizophagus*

**Figure 4:**
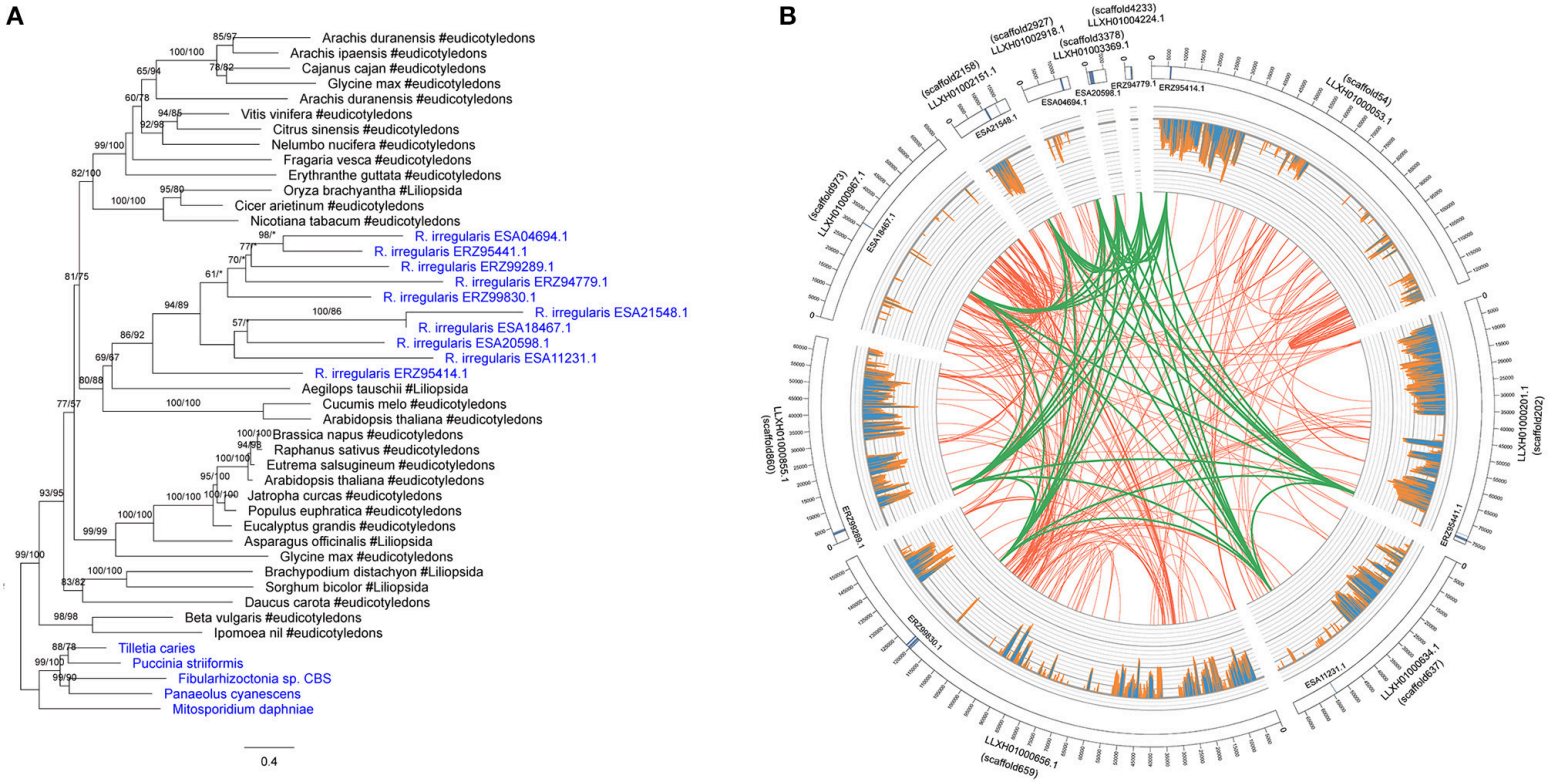
**(A)** Molecular phylogeny of the MULE transposase family. Fungal sequences were obtained from BLASTP output (*E*-value cutoff: 1) and keyword search result. Numbers beside branches represent bootstrap values from maximum likelihood and Bayesian analyses, respectively. Asterisks indicate values lower than 50%. Scale bar represents substitution numbers per amino-acid site. Fungal sequences are colored in blue. **(B)** Circular visualization of the transposase genes mapped on the different scaffolds of *R. irregularis*. Scaffold accession numbers are indicated beside the scaffold bars. The scaffold IDs assigned by Chen et al. (2018) are indicated in parentheses. The sequences maintaining syntenic relationship are linked by lines. Green links: synteny blocks of the transposase genes; Red links: synteny blocks of other segments. The histograms in the middle represent the number of mapped RNA-seq reads.

There were 10 members in the *R. irregularis* MULE transposase gene family (Figure 4A). Multiple gene copies of MULE transposons are not totally unexpected, given their high rates of transposition (Lisch, 2015). All of these 10 copies were only found in various plants, suggesting that they might have been horizontally spread. Gene duplication events were also found in the plants-derived protein kinase gene family (ERZ99026.1 and ESA06945.1) (Supplementary Figure 5) and the bacteria-derived cytotoxin gene family (ESA20238.1 and ERZ96502.1) (Supplementary Figure 26). According to their phylogenetic relationships and domain structures, both families originated from a single HGT event followed by subsequent duplication.

We furthermore explored the syntenic relationship of *R. irregularis* transposase gene family. Because the *R. irregularis* genome assembly released by Tisserant et al. was fragmented (Tisserant et al., 2013), the scaffolds containing acquired genes were short overall (e.g., the longest *R. irregularis* scaffold containing foreign genes was 21844 bp), preventing further structural analyses in this genome. Therefore, we adopted better assembled genomes of four additional *R. irregularis* strains (A1, A4, A5, and C2) (Ropars et al., 2016), and the *R. irregularis* genome released by Chen et al. (2018). Scaffolds with a length <5 kb in above strains were discarded to eliminate unassembled sequences. The acquired genes identified in our study could be mapped to at least one of these genomes (*E*-value cutoff: 1e-10) (Supplementary Table 1). The mapping results (scaffold sequences and anchored positions) based on strain A1 were identical to those based on the genome released by Chen et al. Above results provided additional evidence that the foreign genes identified in our analyses were not contaminations. The draft genome of *R. irregularis* strains A1 (BioProject PRJNA299202), whose N50 was the longest (51,491 bp), was then used as reference to illustrate syntenic relationships of the transposase family. Our analyses indicated that the MULE transposase genes were anchored to 10 different scaffolds in the genome of *R. irregularis* strain A1. Conserved syntenic relationships were observed among these scaffolds (Figure 4B), suggesting duplication occurred in these genomic regions after the acquisition of MULE transposases.

### Expression of acquired genes in *R. irregularis*

To explore the transcriptional activity of the acquired genes in *R. irregularis*, we analyzed previously published transcriptomes of this organism. RNA-seq reads from 41 independent experiments were aligned to the *R. irregularis* genome published by Tisserant et al. (2013). Average fragments per kilobase of transcript per million mapped reads (FPKM) was used to estimate the expression level of *R. irregularis* genes. As a result, 15 out of the 18 acquired genes identified in this study had at least one read mapped onto it, indicating that the vast majority of them are functional under certain conditions (Supplementary Table 2). Nine acquired genes ranked in the top half of the most transcribed genes (i.e., FPKM > 5.40), two of which (ATPase gene: ESA12330.1 and calcium binding protein encoding gene: ESA08389.1) were among the most highly expressed genes (i.e., FPKM > 36.20). These results suggested that ATPase and calcium binding activities might be important for *R. irregularis* in the tested conditions. In addition, six out of the seven acquired genes present in both *R. irregularis* and other fungi (i.e., genes encoding a phosphoglycerate mutase, a prephenate dehydratase, a succinyl-CoA synthetase-like protein, a RNase III, a chromate transporter and a ribokinase), had expression levels between the 42th and 91th percentiles (Supplementary Table 2). Such expression profiles suggested that these six genes might also have necessary functions in these experimental studies.

### Biological processes related to the acquired genes in *R. irregularis*

**Figure 5:**
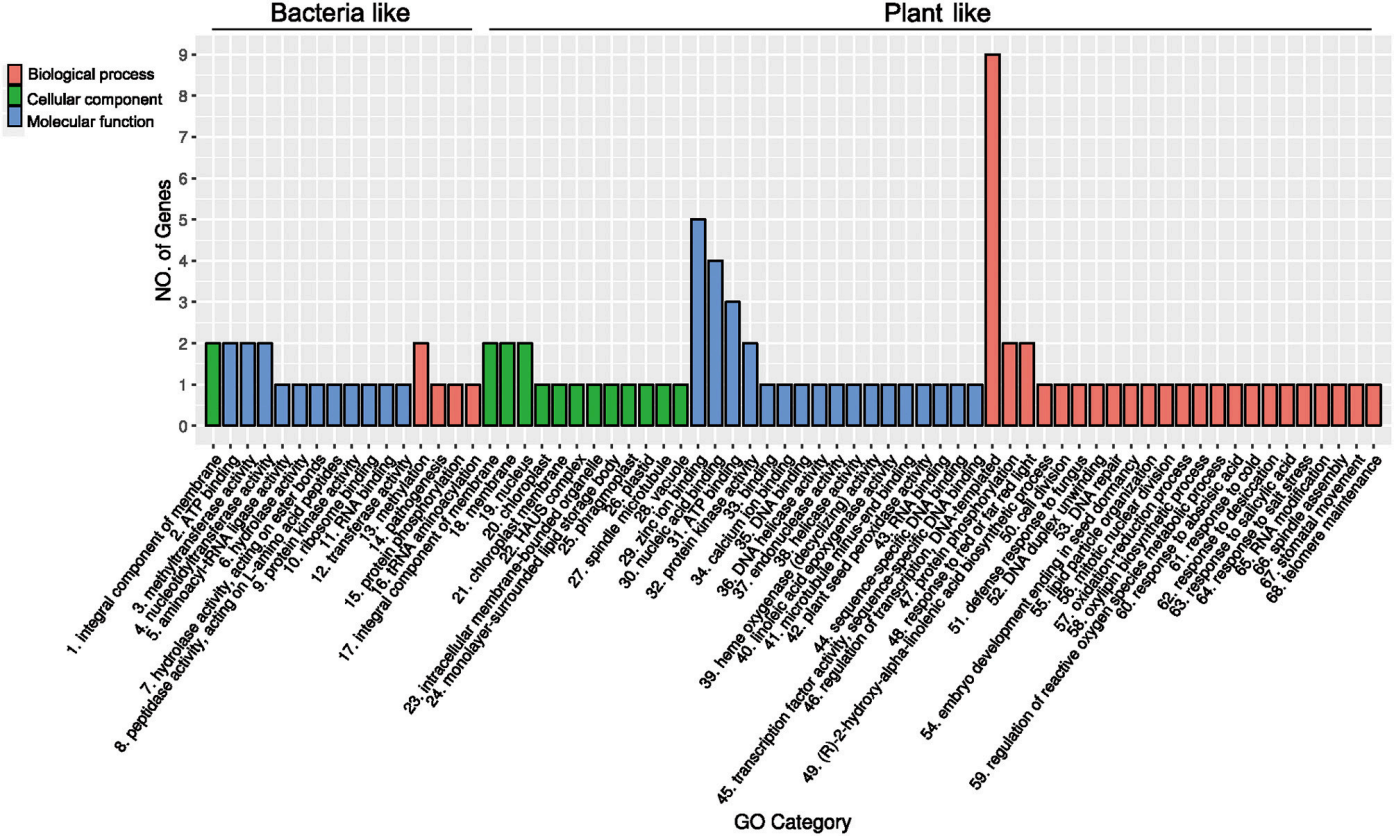
Gene ontology classification of the 18 genes (gene families) recently acquired by *Rhizophagus*.

For a foreign gene to be successfully integrated and retained in the genome of a recipient organism, it would usually need to provide an evolutionary advantage to either the host organism or the gene itself (e.g., selfish genetic elements). Since no prior analyses or experiments exist for the acquired genes in *R. irregularis*, we further investigated their functions based on conserved domains and annotation information. Twelve of these acquired genes contain domains of known function (Table 1). Because some acquired genes were not fully annotated, gene ontology (GO) terms were assigned to them according to their NR matches and InterProScan hits. The distribution of GO terms for these genes is shown in Figure 5 (see Supplementary Table 3 for details). A gene encoding NYN domain containing protein (ESA22410.1) could not be assigned with any GO terms due to the lack of annotation while others contained at least one GO term. For *R. irregularis* genes likely obtained from plants, “regulation of transcription, DNA-templated” from biological processes, and “zinc ion binding” from molecular function were the most abundant GO annotations. It is notable here that both “regulation of transcription” and “zinc ion binding” were encoded by the transposase gene family identified in our analyses, which belongs to FAR1 or MULE domain containing transposable elements (TEs). These selfish genetic elements are common in eukaryotes, and may significantly impact host genome structure, gene expression regulation, signal transduction, and other processes (Eickbush and Malik, 2002; Castanera et al., 2016).

Several genes identified in our analyses encoded RNA binding and processing proteins, including a PPR protein, a nucleotidyltransferase, a NTP_transf_2 domain protein, a NYN domain protein, a class I SAM-dependent methyltransferase and a YbaK/prolyl-tRNA synthetase (Anantharaman and Aravind, 2006; Bartholow et al., 2014; Yamashita et al., 2014; Manna, 2015; Currie et al., 2017). Notably PPR proteins are involved in RNA splicing, editing, processing and translation. These functions are essential for post-transcriptional regulation (Delannoy et al., 2007; Schmitz-Linneweber and Small, 2008; Manna, 2015). Furthermore, two foreign genes, including those encoding HAUS augmin-like complex subunit 5 (ESA01078.1) and polo kinase (ESA11624.1), are functionally related to mitosis. Polo kinases can regulate mitosis by triggering mitotic exit and facilitating removing centromeric cohesin (Mishra et al., 2016; Rodriguez-Rodriguez et al., 2016). The HAUS augmin-like complex plays an important role in mitotic spindle formation, chromosome integrity maintenance, and cytokinesis completion (Goshima et al., 2008; Lawo et al., 2009; Uehara et al., 2009). Additionally, a foreign gene was predicted to encode a calcium-binding protein (caleosin), which is involved in signal transduction and translation pathways (Weidmann et al., 2004).

## Discussion

Whereas vertical inheritance is predominant in eukaryotes, horizontally acquired genes also play an important role in the evolution of many eukaryotic groups (Huang, 2013; Soucy et al., 2015). Some of the acquired genes in eukaryotes are derived from pathogens, host organisms, or prokaryotic endosymbionts (Keeling and Palmer, 2008; Danchin et al., 2010). In this study, we have identified bacteria-derived genes in the genome of *R. irregularis*. The donor bacteria do not belong to known MRE or endobacteria of AMF. One explanation is that *R. irregularis* acquired these bacterial genes directly from other bacteria instead of endobacteria, since mycorrhizal fungi are associated with diverse bacteria (e.g., *Pseudomonas, Burkholderia*, and *Bacillus*) (Bonfante and Anca, 2009; Bonfante and Desiro, 2017). This hypothesis is adequate for *R. irregularis* gene encoding polo kinase, given that it was transferred from *Bacillus* (Xavier and Germida, 2003; Bonfante and Anca, 2009). Considering the fact that genome data of fungal endobacteria are still limited (Torres-Cortes et al., 2015), an alternative explanation is that some unsequenced endobacteria obtained those genes from other bacteria and secondarily transferred them to *R. irregularis*. Both explanations need further evaluation when more endobacterial genomes of AMF become available. Additionally, integration of plant genes in *R. irregularis* implies that genetic information can be transmitted though eukaryote-to-eukaryote endosymbiotic partnerships. To exchange nutrients efficiently, large surface areas between AMF and plant cells are developed by the fungal hyphae penetrating and branching in plant root cells (forming the so-called arbuscules) (Balestrini and Bonfante, 2005; Smith and Smith, 2011). These intracellular interfaces may also serve as an entry point for foreign genes into fungi. In this regard, our results are largely consistent with the “weak-link model,” which suggests that HGT could occur when weakly protected stages (e.g., intracellular arbuscules with thin-walled hyphae) exist in the recipient lifecycle (Huang, 2013).

Our analyses indicated that 18 genes (including three gene families that contain multiple copies) were recently transferred to *R. irregularis* (or its close relatives). The acquisition of RNase III gene from bacteria and PPR gene from plants is consistent with previous researches (Manna, 2015; Lee et al., 2018). Similarly, horizontal transfer of transposase genes in eukaryotes is not surprising, as such transfer events are often considered an important strategy for TEs to escape extinction in their original host lineages (Schaack et al., 2010; El Baidouri et al., 2014; Zhang et al., 2014). Given the stringent approaches (e.g., flanking gene information and strong phylogenetic support) adopted in our analyses, this number is likely to be a minimum estimate of HGTs in *R. irregularis*. Because the available *R. irregularis* genomes were not well assembled (the scaffold N50 of genome released by Tisserant et al. is 5,997 bp; the largest scaffold N50 of genomes released by Ropars et al. and Chen et al. is 51,491 bp), it is possible that some acquired genes were anchored to very short scaffolds, without any flanking genes. Although our strategy can greatly reduce false positives derived from sequencing contamination, some true cases of HGT might have been excluded due to the lack of information on flanking genes. It is also noteworthy that alternative explanations such as differential gene loss, although less parsimonious, remain theoretically possible. This issue of differential gene loss may be further complicated by the limited high-quality genome data available for protists. As such, the origin of genes identified in our analyses need to be evaluated when more genomes are sequenced and accurately assembled.

Gene family expansion following the acquisition of foreign genes, including genes encoding MULE transposase, protein kinase and cytotoxin, has been found in *R. irregularis*. These three gene families might have originated from a HGT event followed by duplication events. We also found that more than half of the foreign genes were expressed in transcriptome experiments. A previous study suggested that high gene expression levels often had a negative impact on gene transferability (Park and Zhang, 2012). Nevertheless, our results suggest that the acquired genes in *R. irregularis* have been fixed, and are capable of providing beneficial phenotypes. Indeed, one of the top transcribed foreign genes in tested conditions was predicted to encode a calcium-binding protein, which is important in signal transduction and translation (Weidmann et al., 2004). Calcium-binding proteins in fungi are also associated with conidial germination, lipid storage and infection ability (Fan et al., 2015; Hanano et al., 2015; Ortiz-Urquiza et al., 2016). Similarly, calcium-binding proteins in plants not only participate in signal transduction (Shen et al., 2014), but also act as a structural protein of lipid bodies (Partridge and Murphy, 2009; Jiang and Tzen, 2010; Shimada et al., 2015). More specifically for arbuscular mycorrhizae, calcium-binding proteins participate in calcium-modulated signaling pathways that can induce interactions between AMF and plant roots (Liu et al., 2013). This result indicates that the acquisition of calcium-binding protein gene might have facilitated *R. irregularis*-plants symbiosis.

Functional analyses showed that acquired genes in *R. irregularis* might be associated with spore and hyphal growth. Particularly, the acquired genes in *R. irregularis* include genes encoding polo kinase and HAUS augmin-like protein, both of which regulate mitosis progression. For example, during mitosis, polo kinase promotes removing of centromeric cohesin, and HAUS augmin-like protein is involved in mitotic spindle formation (Goshima et al., 2008; Mishra et al., 2016). In *Arabidopsis*, augmin participates in organization of the spindle during cell division (Ho et al., 2011). In the fungus *Aspergillus nidulans*, although its function remains unclear, augmin is enriched at the spindle pole body and binds to spindle microtubules during mitosis (Edzuka et al., 2014). Mitosis is involved in fungal spore formation and hyphal growth (Horio and Oakley, 2005; Marleau et al., 2011). Under high-phosphate conditions, not only is mitosis repressed, but the development of arbuscular mycorrhizae is strongly suppressed as well (Sugimura and Saito, 2017). Given the role of polo kinase and HAUS augmin-like protein in mitosis, acquisition of these two genes might have contributed to the development of *R. irregularis*.

Our results also show that acquired genes in *R. irregularis* likely contribute to the regulation of gene expression through post-transcriptional and post-translational modifications (Mata et al., 2005). For instance, PPR proteins can bind to precursor RNAs via the PPR modules and this process is required for proper splicing (Schmitz-Linneweber and Small, 2008). Likewise, NYN domain containing proteins maintain ribonuclease activity that affects RNA-processing (Anantharaman and Aravind, 2006). In addition, methyltransferases are involved in transcriptional activation and silencing (Lyko, 2018). Although direct evidence for the involvement of these genes in symbiosis is lacking, there is no doubt that transcriptional regulation of different gene expression is necessary for various biological processes, including the establishment of AMF-plants symbiosis (Liu et al., 2003; Lanfranco et al., 2005; Balestrini and Lanfranco, 2006; Tsuzuki et al., 2016). Whether and how these acquired genes may control symbiosis stage-specific gene expression needs further experimental investigations.

## Author contributions

JH conceived the research, ML performed analyses, JH and ML wrote the manuscript, JZ and NT participated in data interpretation and manuscript revision, HS helped with analyses.

### Conflict of interest statement

The authors declare that the research was conducted in the absence of any commercial or financial relationships that could be construed as a potential conflict of interest.

## Supplementary material

Supplementary Table 1 Results of mapping *R. irregularis* proteins (released by Tisserant et al.) to genomes of four other strains.

Supplementary Table 2 Expression profile of *R. irregularis* genes.

Supplementary Table 3 GO terms of *R. irregularis* foreign genes.

Supplementary File 1 This file contains Supplementary Methods and Supplementary Figures 1–26.
