# Supplementary material for: Horizontal Gene Transfer From Bacteria and Plants to the Arbuscular Mycorrhizal Fungus Rhizophagus irregularis
Source: Front Plant Sci. 2018 May 25;9:701. doi: 10.3389/fpls.2018.00701 (PMC5982333; doi:10.3389/fpls.2018.00701)
Supplement: Supplementary File 1 — This file contains Supplementary Methods and Supplementary Figures 1–26. [file Data_Sheet_1.PDF]

Supplementary Material

Horizontal gene transfer from bacteria and plants to the arbuscular mycorrhizal fungus *Rhizophagus irregularis*

Meng Li, Jinjie Zhao, Nianwu Tang, Hang Sun*, Jinling Huang*

*** Correspondence:**

Hang Sun

sunhang@mail.kib.ac.cn

Jinling Huang

huangj@ecu.edu

Supplemental Methods

During the phylogenetic analyses, the RefSeq/NR sampling covers bacteria, archaebacterial, viruses, green plants, fungi and other eukaryotes. Each of these groups was divided into subgroups. We selected hits from these subgroups (E value cutoff: 1e-3), one hit per species, and at most 10 hits per group. To gain sufficient and balanced samplings from each representative taxa, manual inspection and additional samplings would be performed when necessary. If additional fungal/plant sequences were required and there were no more BLAST hits with E value cutoff of 1e-3, BLAST hits with E value < 1 would be considered and keyword search would be performed. If a HGT event won't be affected even with fewer sequences from a group (according to ML analyses), the number of samplings from that group would be reduced to save computing resources (Bayesian analyses were extremely time consuming). The detailed group information were listed as follows (NCBI taxonomy ID and names):

Bacteria: 1236: Gammaproteobacteria; 28211: Alphaproteobacteria; 28216: Betaproteobacteria; 32045: unclassified Proteobacteria; 68525: delta.epsilon subdivisions; 580370: Zetaproteobacteria; 1553900: Oligoflexia; 1807140: Acidithiobacillia; 1817794: Candidatus Lambdaproteobacteria; 1817795: Candidatus Muproteobacteria; 2323: unclassified Bacteria; 32066: Fusobacteria; 40117: Nitrospirae; 57723: Acidobacteria; 67814: Caldiserica; 68297: Dictyoglomi; 74152: Elusimicrobia; 200783: Aquificae; 200918: Thermotogae; 200930: Deferribacteres; 200938: Chrysiogenetes; 200940: Thermodesulfobacteria; 203691: Spirochaetes; 508458: Synergistetes; 1783257: PVC group; 1783270: FCB group; 1239: Firmicutes; 1297: Deinococcus-Thermus; 67819: Armatimonadetes; 200795: Chloroflexi; 201174: Actinobacteria; 544448: Tenericutes; 1859062: unclassified Terrabacteria; 1117: Cyanobacteria; 1798710: Candidatus Melainabacteria; 1919278: unclassified Cyanobacteria.Melainabacteria; 1802340: Nitrospinae.Tectomicrobia; 1853220: Rhodothermaeota; 1930617: Calditrichaeot;

Fungi: 4761: Chytridiomycota; 6029: Microsporidia; 89443: unclassified Fungi; 112252: Fungi incertae sedis; 451455: Neocallimastigomycota; 451459: Blastocladiomycota; 4890: Ascomycota; 5204: Basidiomycota; 1210850: unclassified Dikarya; 1696033: Entorrhizomycota; 584652: Mixed fungal DNA libraries; 1031332: Cryptomycota; 214506: Glomeromycetes; 241590: unclassified Glomeromycota; 451507: Mucoromycotina; 1137986: Mortierellomycotina; 1913638: Zoopagomycota;

Viruses: 12333: unclassified phages; 12429: unclassified viruses; 12877: Satellites; 29258: ssDNA viruses; 35237: dsDNA viruses.no RNA stage; 35268: Retro_transcribing viruses; 35325: dsRNA viruses; 39759: Deltavirus; 439488: ssRNA viruses; 451344: unclassified archaeal viruses; 552364: unclassified virophages; 686617: unassigned viruses; 1425366: Virus-associated RNAs;

Archaea: 183925: Methanobacteria; 183939: Methanococci; 183963: Halobacteria; 183967: Thermoplasmata; 183968: Thermococci; 183980: Archaeoglobi; 183988: Methanopyri; 33867: unclassified Euryarchaeota; 224756: Methanomicrobia; 1775750: Hadesarchaea; 29294: unclassified Archaea; 1783275: TACK group; 1783276: DPANN group;

Green plants: 3041: Chlorophyta; 96475: Mesostigmatophyceae; 131213: Chlorokybophyceae; 131220: Klebsormidiophyceae; 131209: Zygnemophyceae; 304573: Coleochaetophyceae; 304574: Charophyceae; 3195: Marchantiophyta; 3208: Bryophyta; 13809: Anthocerotophyta; 42898: unclassified Embryophyta; 1521260: Lycopodiidae; 241806: Moniliformopses; 1392478: unclassified Spermatophyta; 1437180: Acrogymnospermae; 232365: basal Magnoliophyta; 232379: unclassified Magnoliophyta; 4447: Liliopsida; 71240: eudicotyledons; 91811: Ceratophyllales; 232347: Magnoliidae; 261008: Chloranthales;

Other groups: 12884: Viroids; 2763: Rhodophyta; 2830: Haptophyceae; 3027: Cryptophyta; 5719: Parabasalia; 5752: Heterolobosea; 33630: Alveolata; 33634: Stramenopiles; 33682: Euglenozoa; 38254: Glaucocystophyceae; 42452: unclassified eukaryotes; 66288: Oxymonadida; 136087: Malawimonadidae; 193537: Centroheliozoa; 207245: Fornicata; 339960: Katablepharidophyta; 543769: Rhizaria; 554296: Apusozoa; 554915: Amoebozoa; 556282: Jakobida; 1401294: Breviatea; 28009: Choanoflagellida; 33208: Metazoa; 42461: Opisthokonta incertae sedis; 1001604: Nucleariidae Fonticula group.

Supplemental Figures

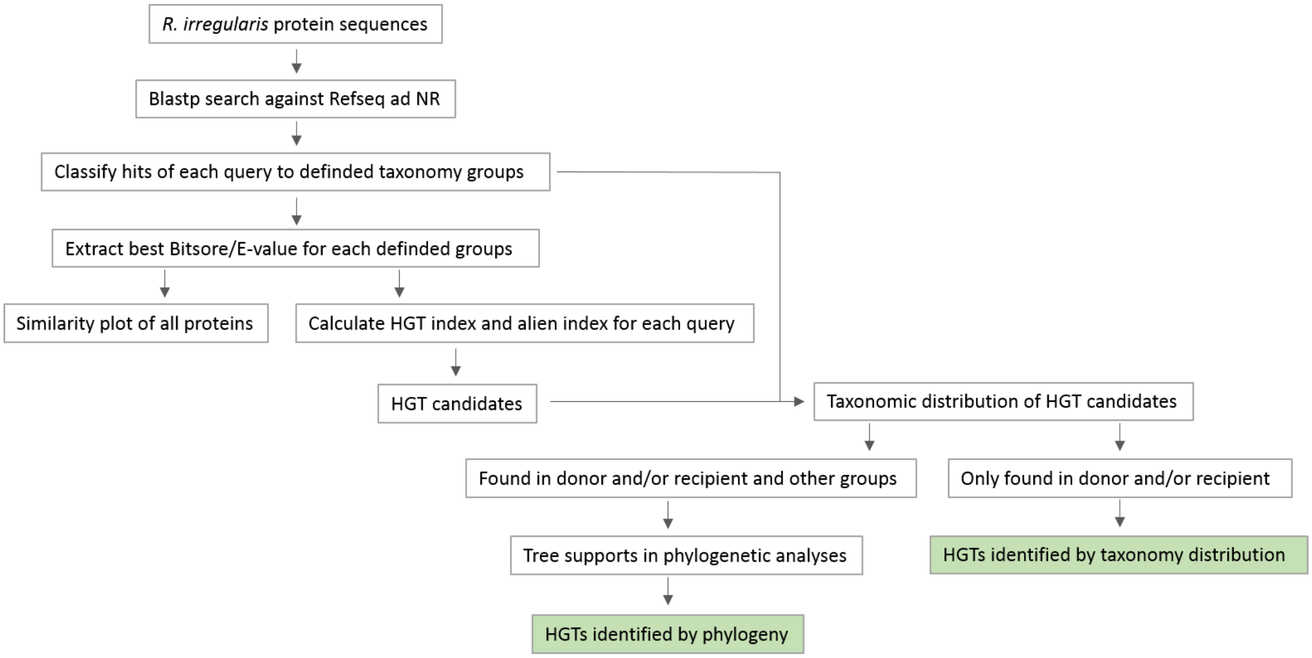

Supplementary Figure 1. The flowchart of HGT detection process in this study.

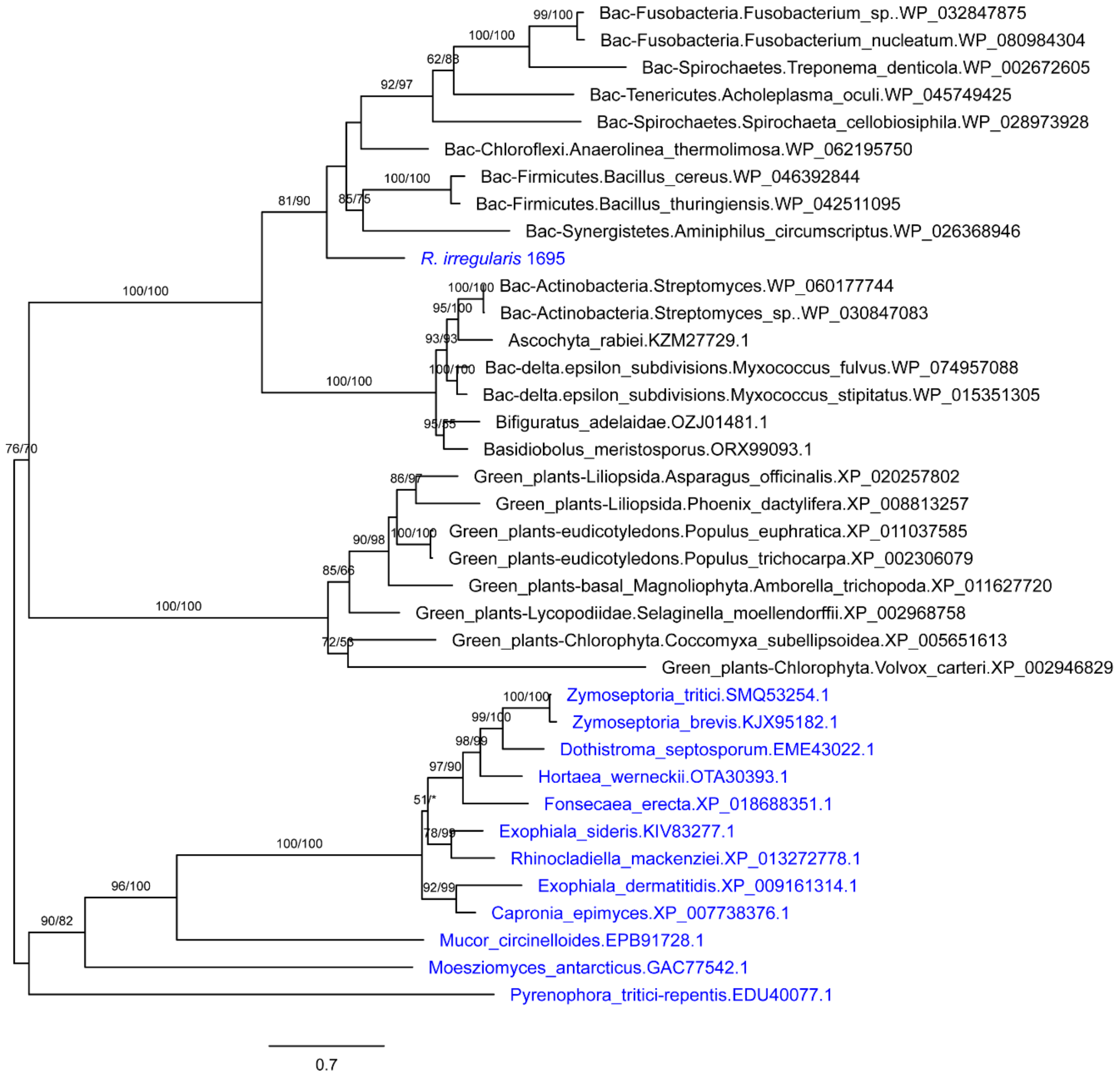

Supplementary Figure 2 Molecular phylogeny of class I SAM-dependent methyltransferases. Fungal sequences were obtained from BLASTP output (E value cutoff: 1e-3) and keyword search result. Numbers beside branches represent bootstrap values from maximum likelihood and Bayesian results, respectively. Asterisks indicate values lower than 50%. Scale bars represent substitution numbers per amino-acid site. Fungal sequences are colored in blue.

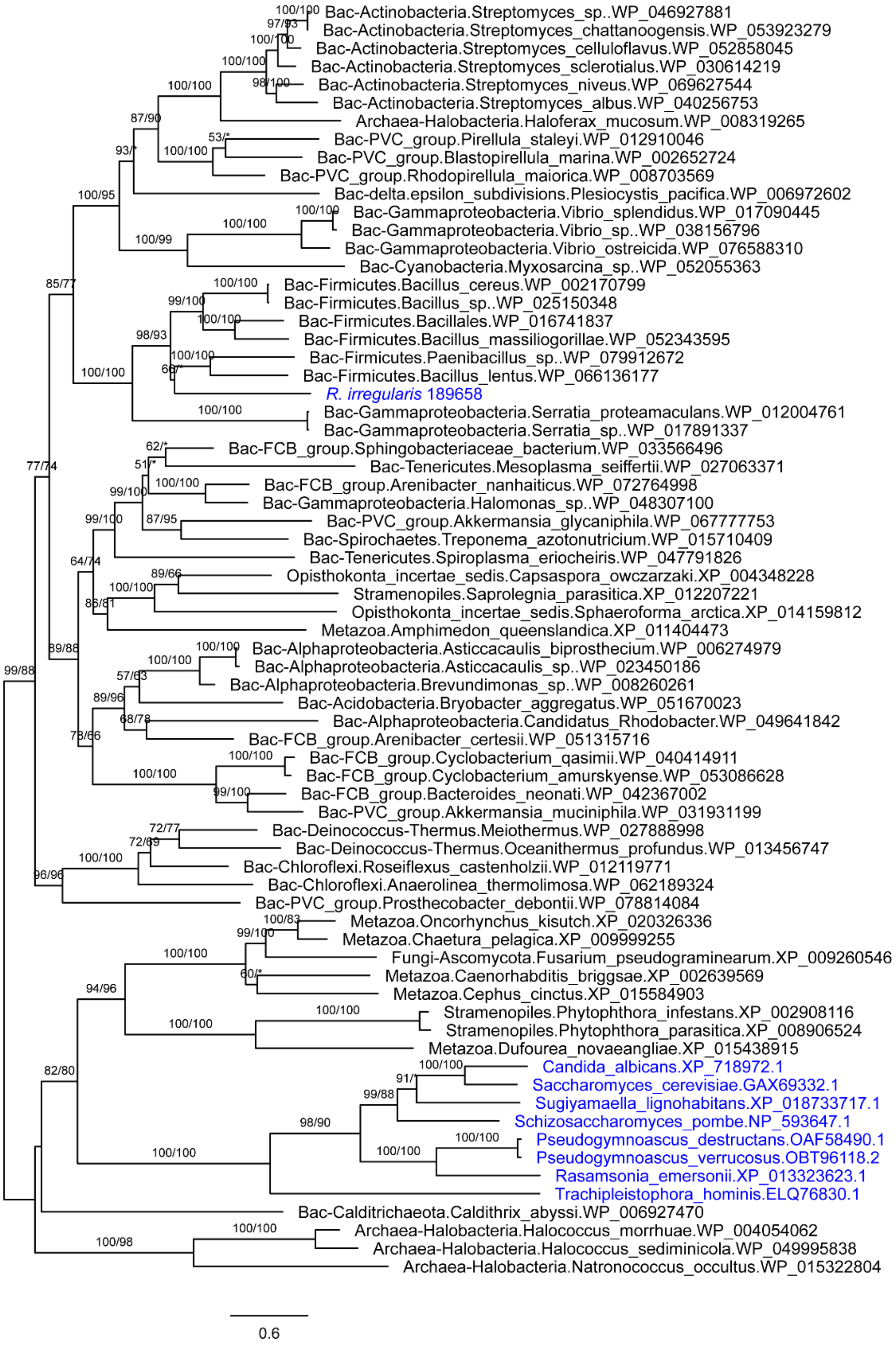

Supplementary Figure 3 Molecular phylogeny of polo kinases. Fungal sequences were obtained from BLASTP output (E value cutoff: 1e-3) and keyword search result. Numbers beside branches represent bootstrap values from maximum likelihood and Bayesian results, respectively. Asterisks indicate values lower than 50%. Scale bars represent substitution numbers per amino-acid site. Fungal sequences are colored in blue.

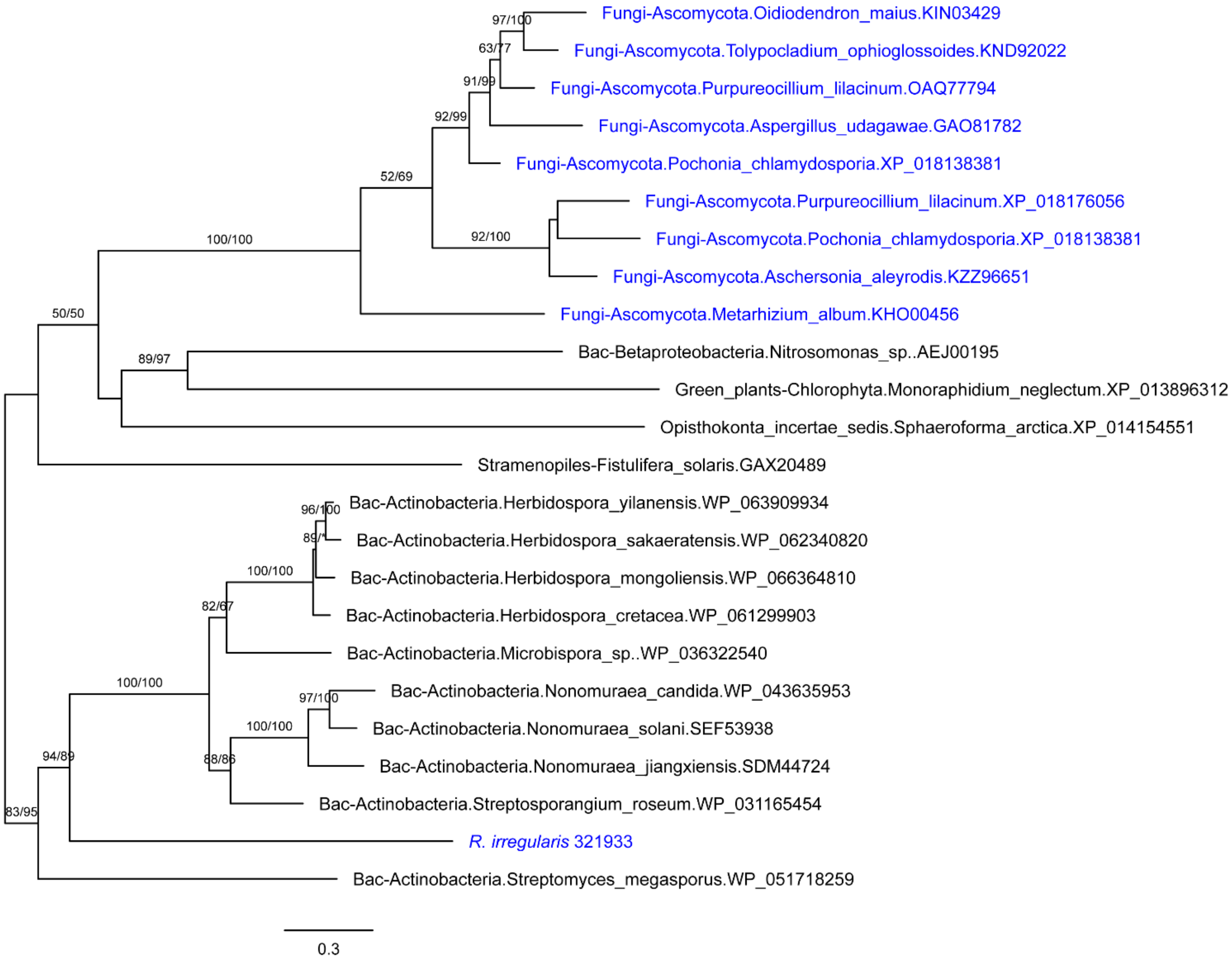

Supplementary Figure 4 Molecular phylogeny of hypothetical protein ESA16208.1 (321933). Fungal sequences were obtained from BLASTP output (E value cutoff: 1e-3). Numbers beside branches represent bootstrap values from maximum likelihood and Bayesian results, respectively. Asterisks indicate values lower than 50%. Scale bars represent substitution numbers per amino-acid site. Fungal sequences are colored in blue.

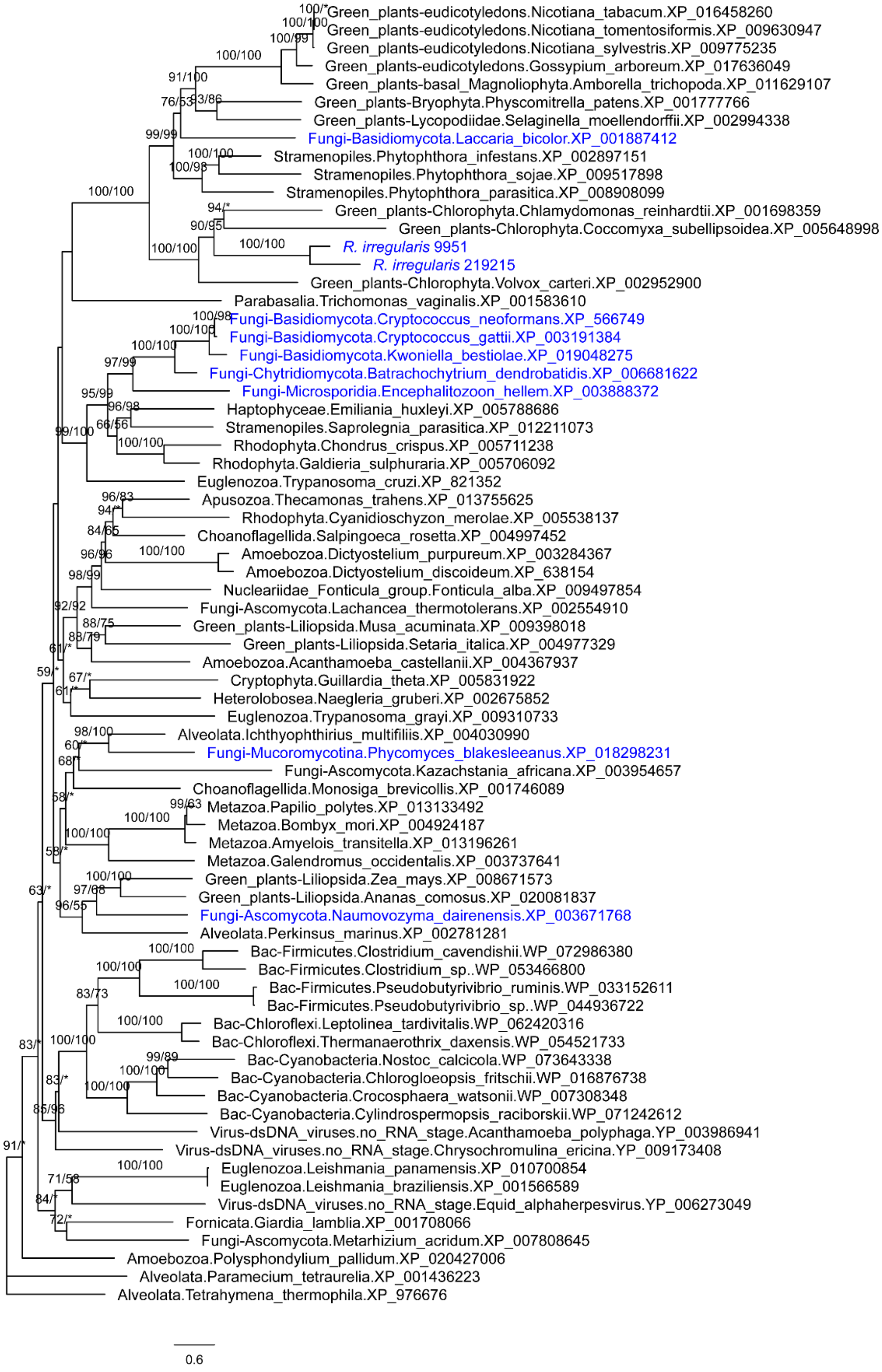

Supplementary Figure 5 Molecular phylogeny of protein kinases. Fungal sequences were obtained from BLASTP output (E value cutoff: 1e-3). Numbers beside branches represent bootstrap values from maximum likelihood and Bayesian results, respectively. Asterisks indicate values lower than 50%. Scale bars represent substitution numbers per amino-acid site. Fungal sequences are colored in blue.

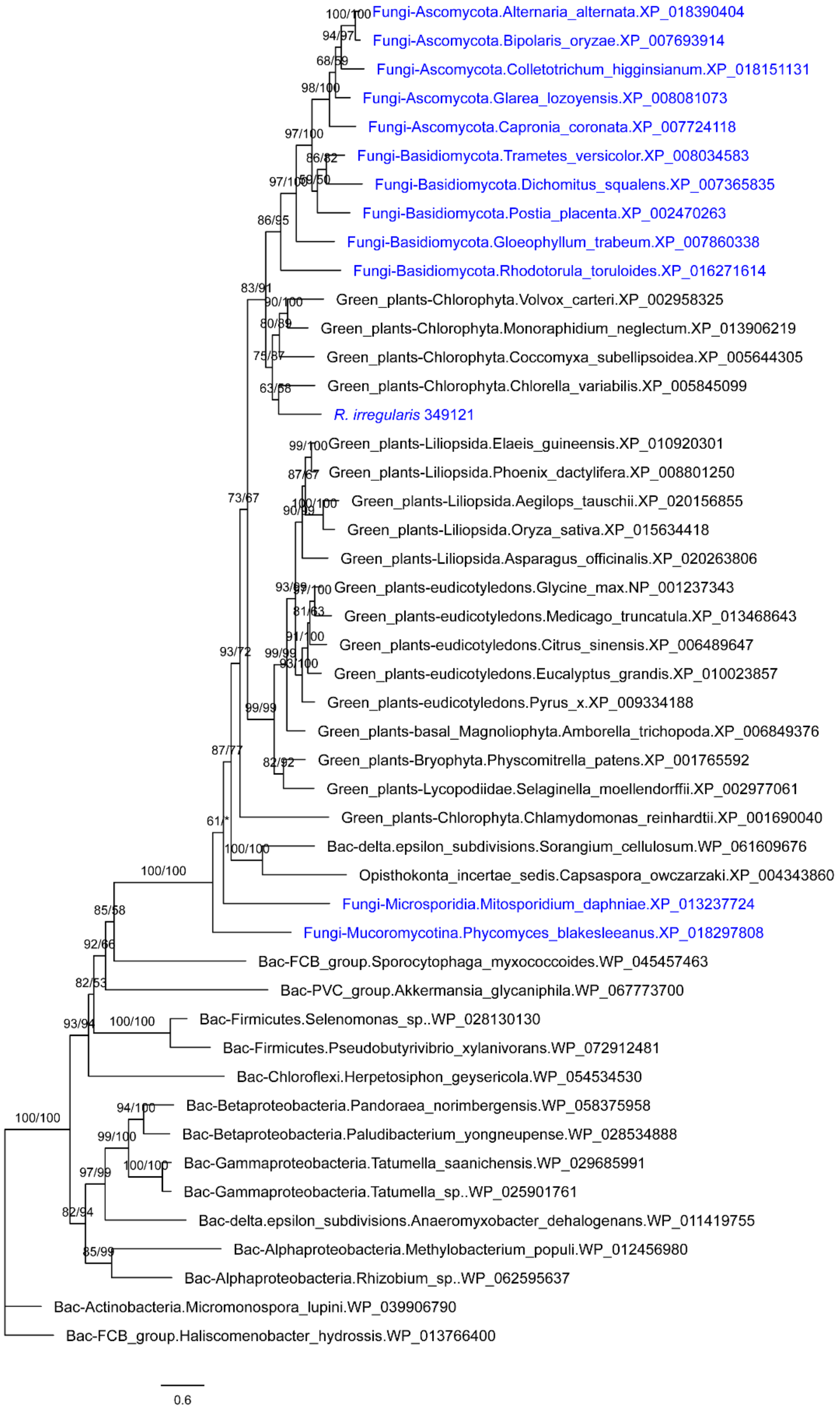

Supplementary Figure 6 Molecular phylogeny of calcium binding proteins. Fungal sequences were only obtained from BLASTP output (E value cutoff: 1e-3). Numbers beside branches represent bootstrap values from maximum likelihood and Bayesian results, respectively. Asterisks indicate values lower than 50%. Scale bars represent substitution numbers per amino-acid site. Fungal sequences are colored in blue.

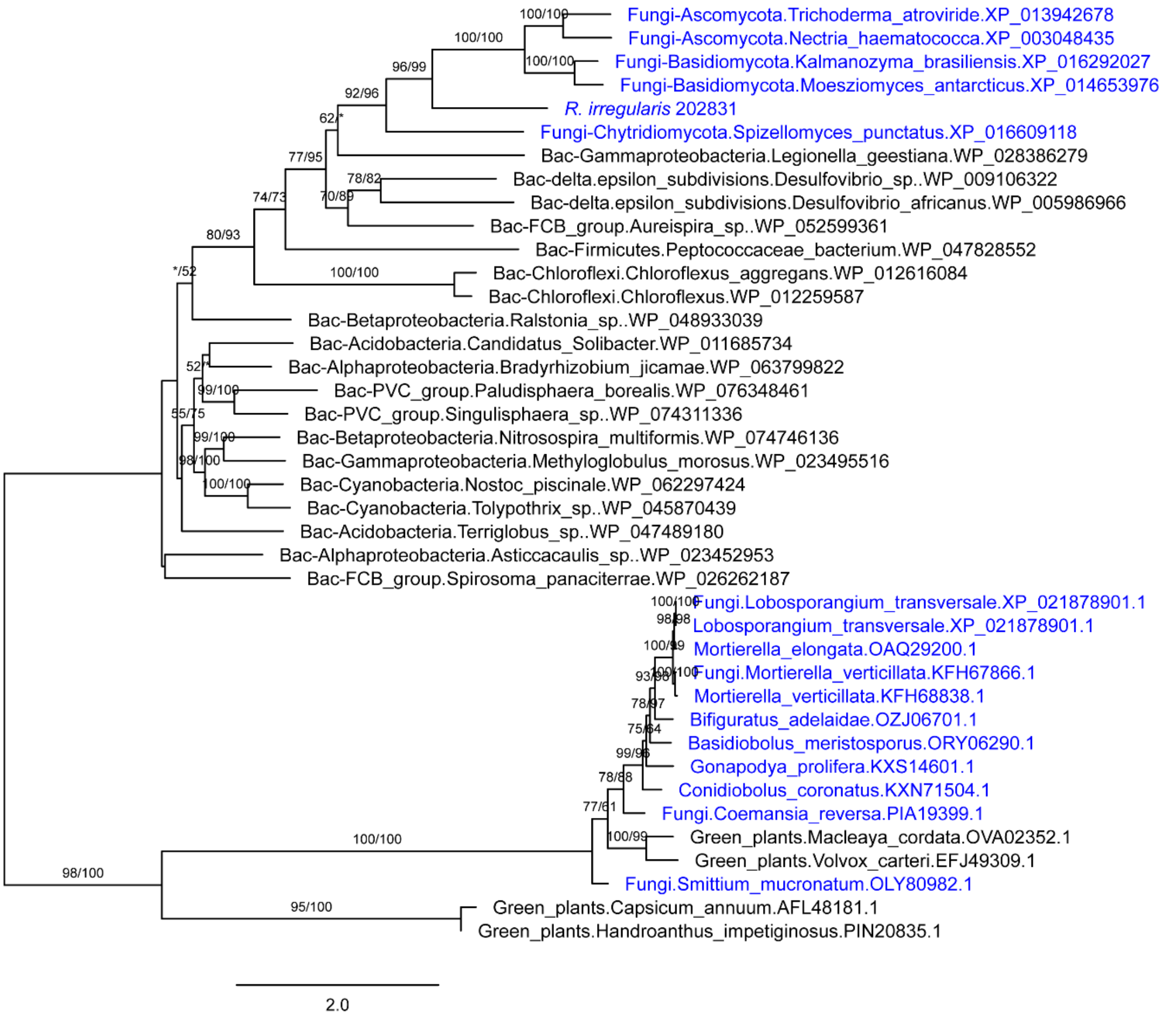

Supplementary Figure 7 Molecular phylogeny of succinyl-CoA synthetases. Fungal sequences were obtained from BLASTP output (E value cutoff: 1e-3) and keyword search result. Numbers beside branches represent bootstrap values from maximum likelihood and Bayesian results, respectively. Asterisks indicate values lower than 50%. Scale bars represent substitution numbers per amino-acid site. Fungal sequences are colored in blue.

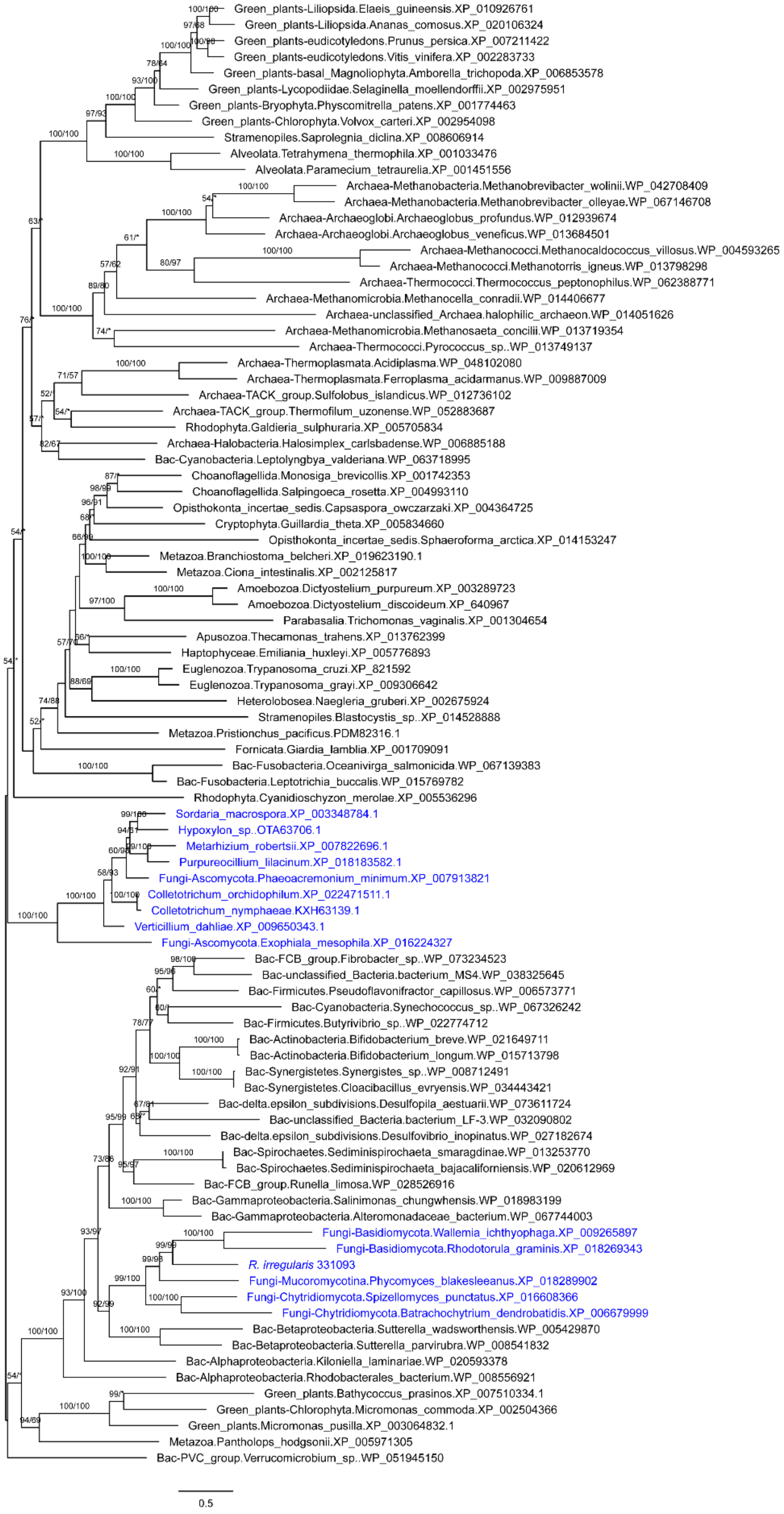

Supplementary Figure 8 Molecular phylogeny of ribokinases. Fungal sequences were obtained from BLASTP output (E value cutoff: 1e-3) and keyword search result. Numbers beside branches represent bootstrap values from maximum likelihood and Bayesian results, respectively. Asterisks

indicate values lower than 50%. Scale bars represent substitution numbers per amino-acid site. Fungal sequences are colored in blue.

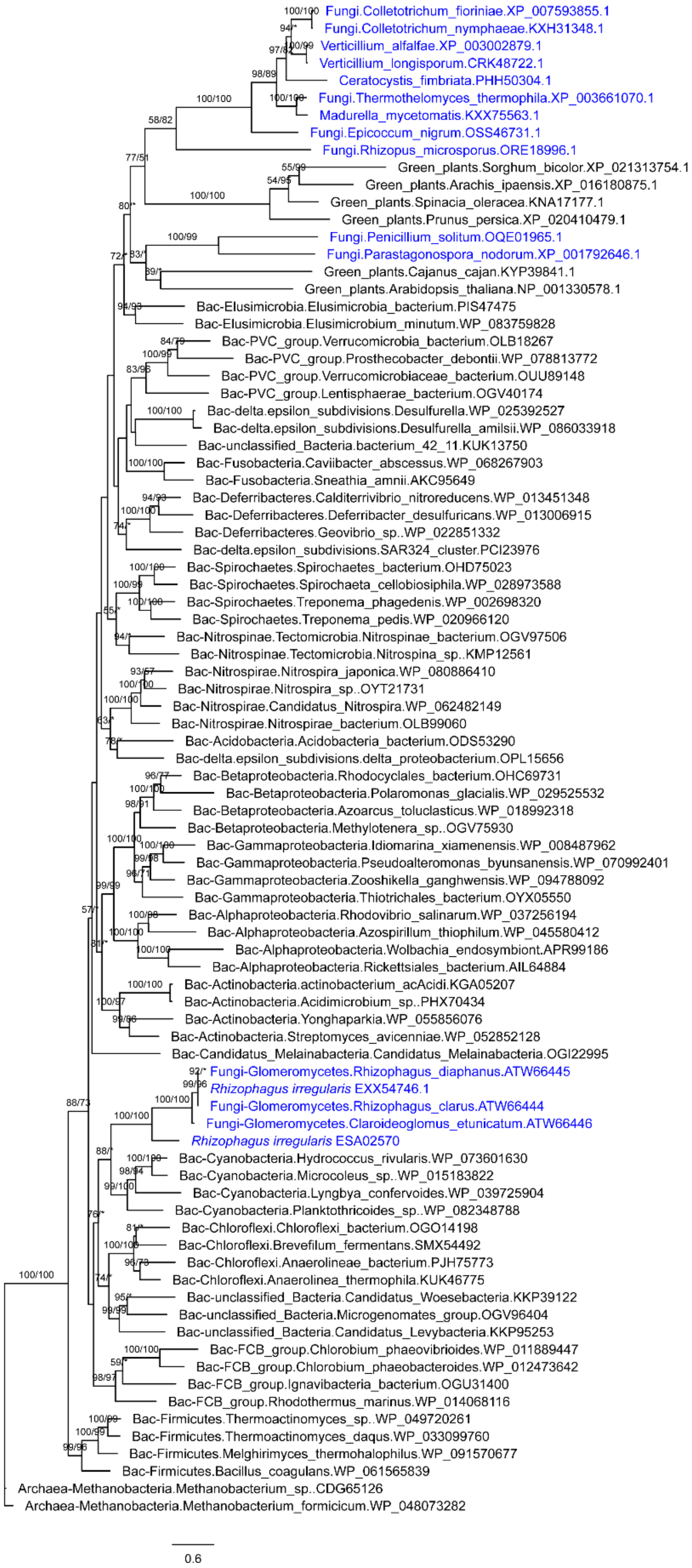

Supplementary Figure 9 Molecular phylogeny of RNase IIIs. Fungal sequences were obtained from BLASTP output (E value cutoff: 1e-3) and keyword search result. Numbers beside branches represent bootstrap values from maximum likelihood and Bayesian results, respectively. Asterisks

indicate values lower than 50%. Scale bars represent substitution numbers per amino-acid site. Fungal sequences are colored in blue.

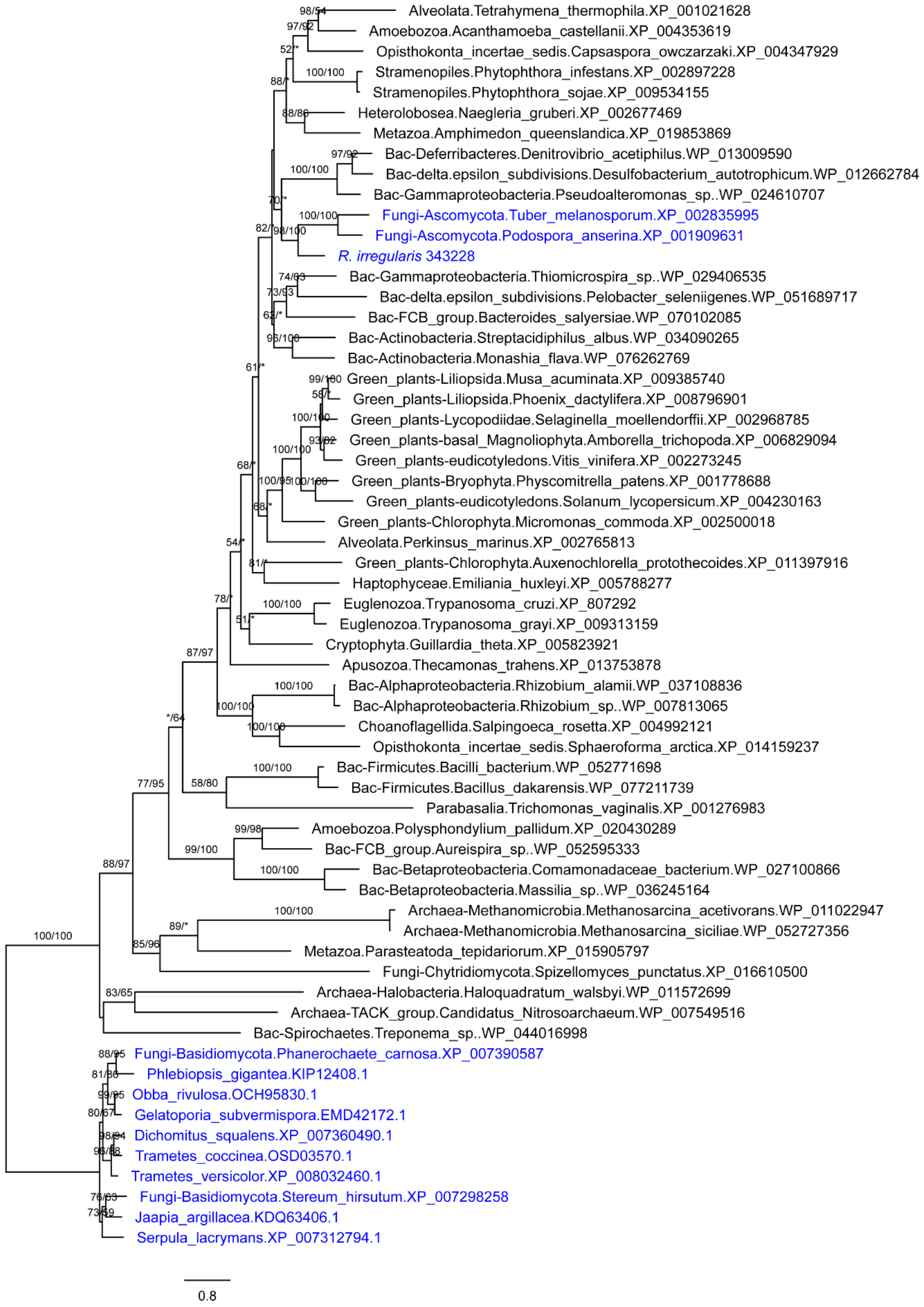

Supplementary Figure 10 Molecular phylogeny of phosphoglycerate mutases. Fungal sequences were obtained from BLASTP output (E value cutoff: 1e-3) and keyword search result. Numbers

beside branches represent bootstrap values from maximum likelihood and Bayesian results, respectively. Asterisks indicate values lower than 50%. Scale bars represent substitution numbers per amino-acid site. Fungal sequences are colored in blue.

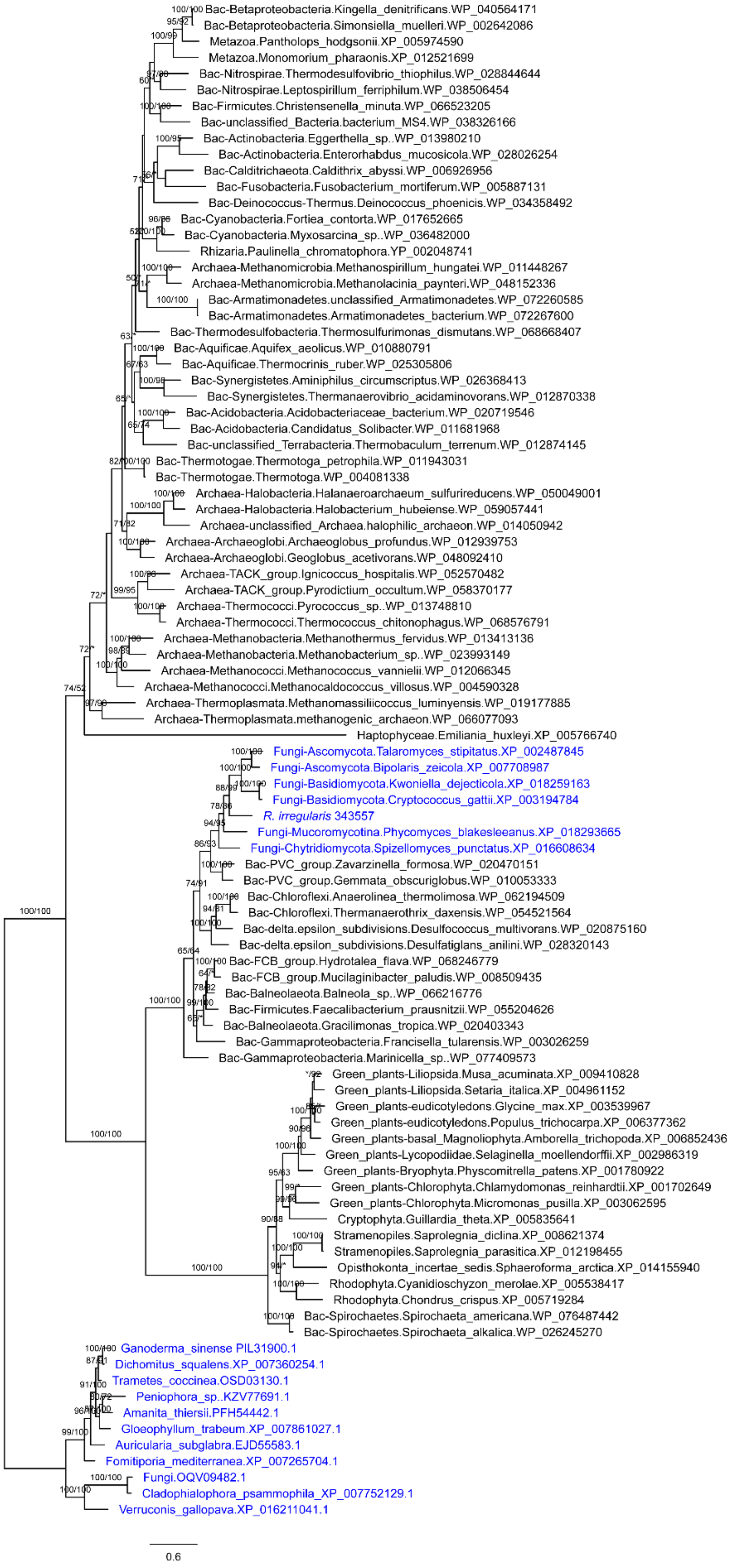

Supplementary Figure 11 Molecular phylogeny of ketol-acid reductoisomerases. Fungal sequences were obtained from BLASTP output (E value cutoff: 1e-3) and keyword search result. Numbers beside branches represent bootstrap values from maximum likelihood and Bayesian results,

respectively. Asterisks indicate values lower than 50%. Scale bars represent substitution numbers per amino-acid site. Fungal sequences are colored in blue.

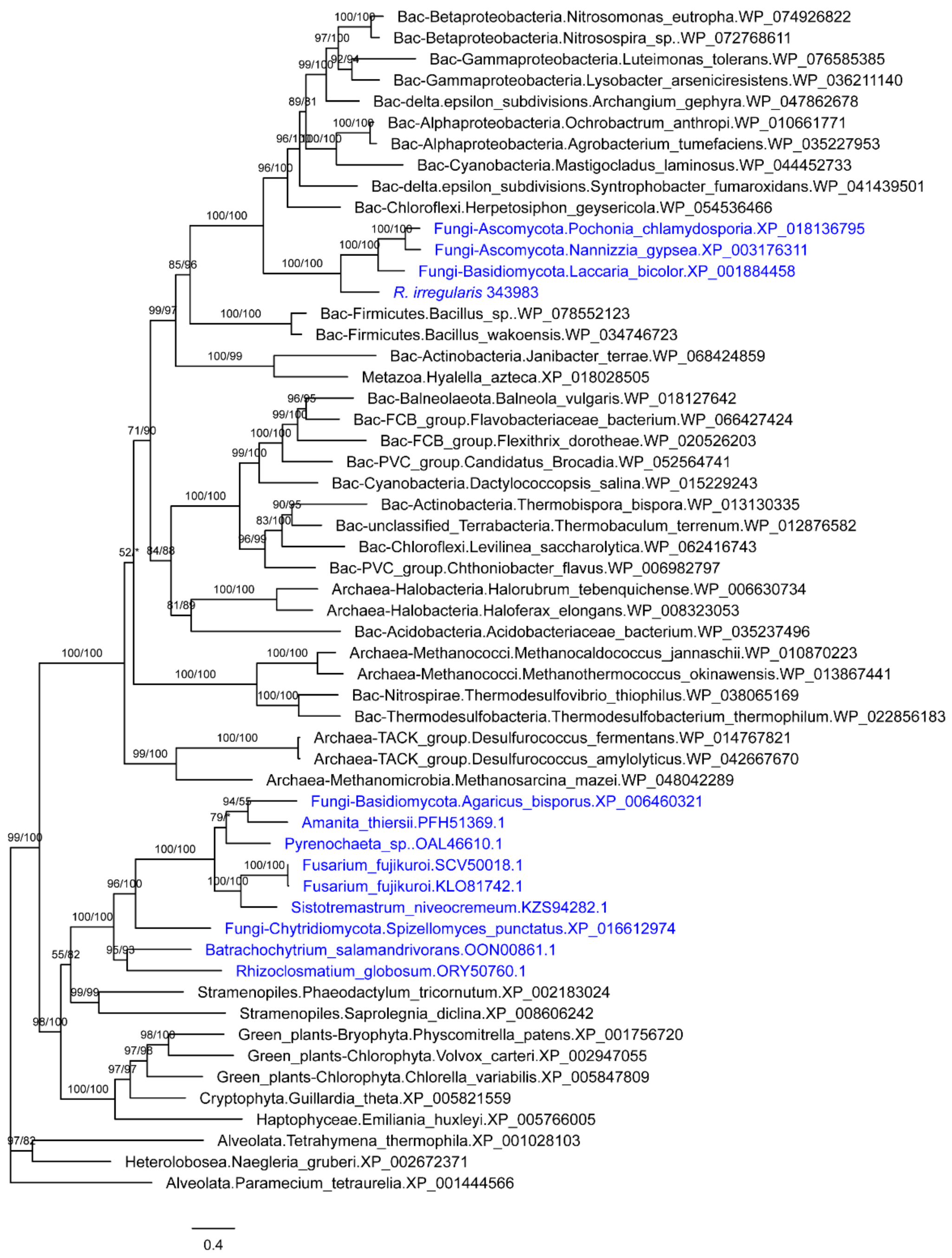

Supplementary Figure 12 Molecular phylogeny of chromate transporters. Fungal sequences were obtained from BLASTP output (E value cutoff: 1e-3) and keyword search result. Numbers beside branches represent bootstrap values from maximum likelihood and Bayesian results, respectively. Asterisks indicate values lower than 50%. Scale bars represent substitution numbers per amino-acid site. Fungal sequences are colored in blue.

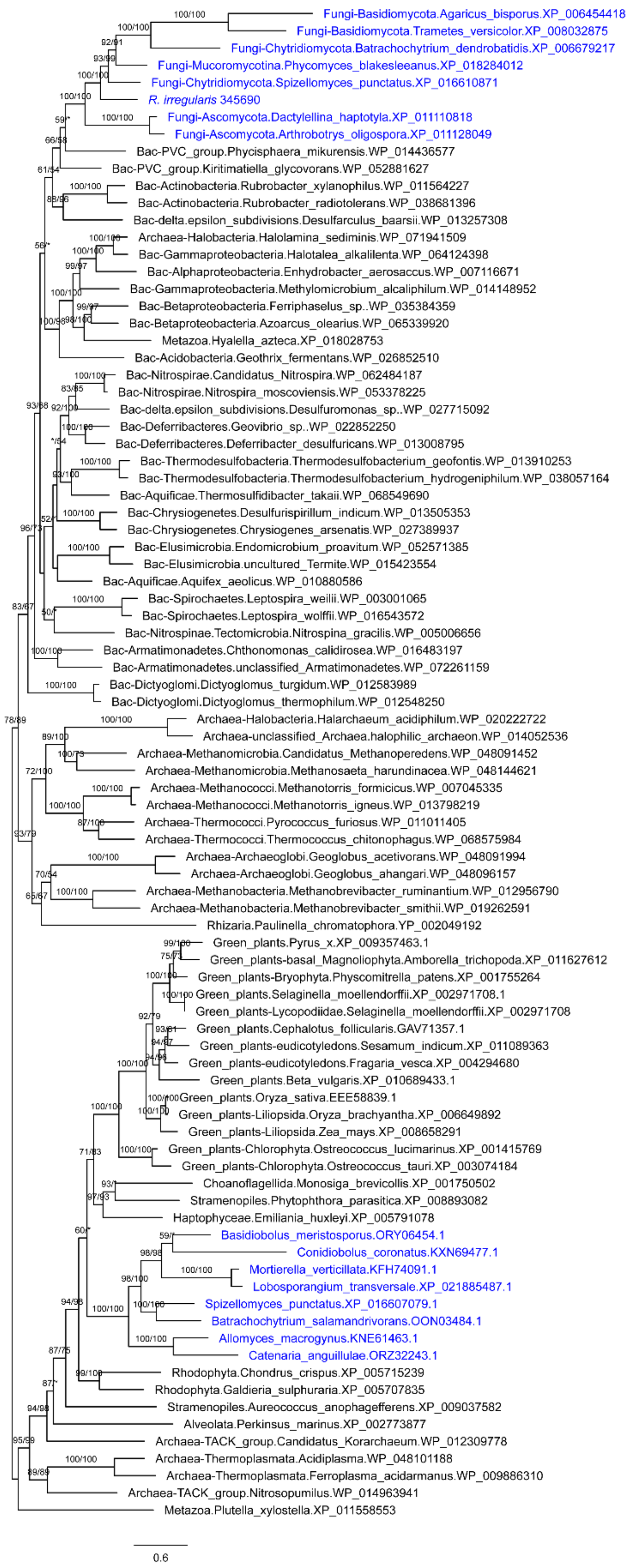

Supplementary Figure 13 Molecular phylogeny of prephenate dehydratases. Fungal sequences were obtained from BLASTP output (E value cutoff: 1e-3) and keyword search result. Numbers beside branches represent bootstrap values from maximum likelihood and Bayesian results, respectively.

Asterisks indicate values lower than 50%. Scale bars represent substitution numbers per amino-acid site. Fungal sequences are colored in blue.

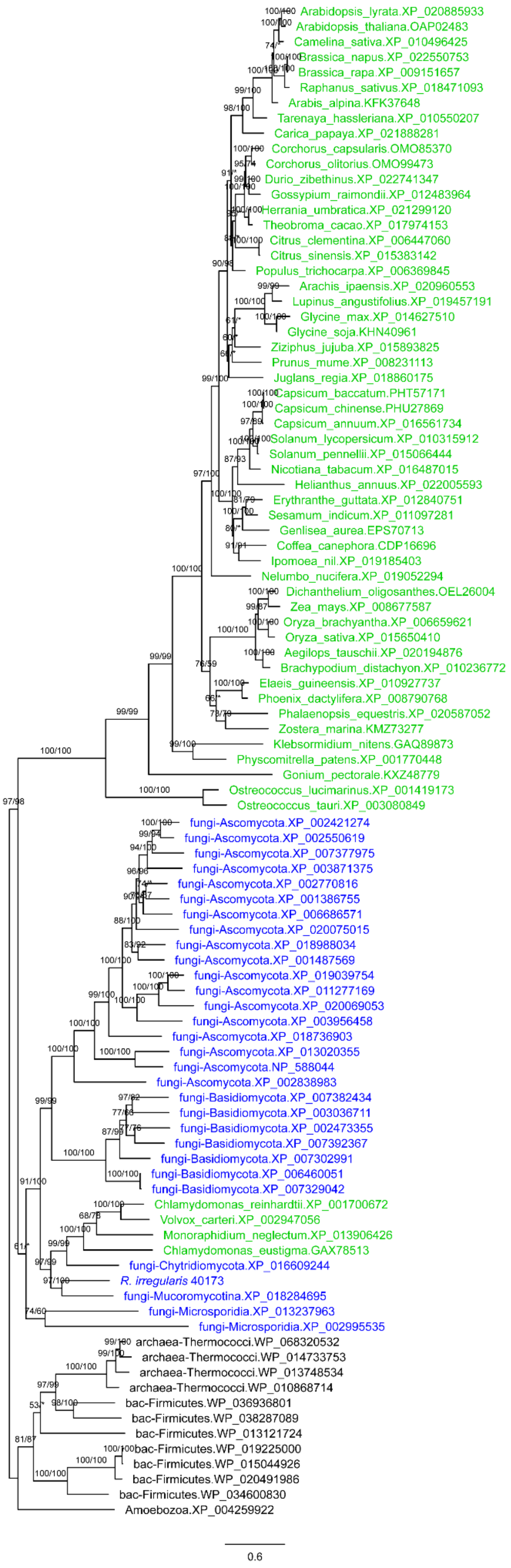

Arabidopsis_lyrata.XP_020885933
Arabidopsis_thaliana.OAP02483
Camelina_sativa.XP_010496425
Brassica_napus.XP_022550753
Brassica_rapa.XP_009151657
Raphanus_sativus.XP_018471093
Arabis_alpina.KFK37648
Tarenaya_hassleriana.XP_010550207
Carica_papaya.XP_021888281
Corchorus_capsularis.OMO85370
Corchorus_olitorius.OMO99473
Durio_zibethinus.XP_022741347
Gossypium_raimondii.XP_012483964
Herrania_umbratica.XP_021299120
Theobroma_cacao.XP_017974153
Citrus_clementina.XP_006447060
Citrus_sinensis.XP_015383142
Populus_trichocarpa.XP_006369845
Arachis_ipaensis.XP_020960553
Lupinus_angustifolius.XP_019457191
Glycine_max.XP_014627510
Glycine_soja.KHN40961
Ziziphus_jujuba.XP_015893825
Prunus_mume.XP_008231113
Juglans_regia.XP_018860175
Capsicum_baccatum.PHT57171
Capsicum_chinense.PHU27869
Capsicum_annuum.XP_016561734
Solanum_lycopersicum.XP_010315912
Solanum_pennellii.XP_015066444
Nicotiana_tabacum.XP_016487015
Helianthus_annuus.XP_022005593
Erythranthe_guttata.XP_012840751
Sesamum_indicum.XP_011097281
Genlisea_aurea.EPS70713
Coffea_canephora.CDP16696
Ipomoea_nil.XP_019185403
Nelumbo_nucifera.XP_019052294
Dichanthelium_oligosanthes.OEL26004
Zea_mays.XP_008677587
Oryza_brachyantha.XP_006659621
Oryza_sativa.XP_015650410
Aegilops_tauschii.XP_020194876
Brachypodium_distachyon.XP_010236772
Elaeis_guineensis.XP_010927737
Phoenix_dactylifera.XP_008790768
Phalaenopsis_equestris.XP_020587052
Zostera_marina.KMZ73277
Klebsormidium_nitens.GAQ89873
Physcomitrella_patens.XP_001770448
Gonium_pectorale.KXZ48779
Ostreococcus_lucimarinus.XP_001419173
Ostreococcus_tauri.XP_003080849
fungi-Ascomycota.XP_002421274
fungi-Ascomycota.XP_002550619
fungi-Ascomycota.XP_007377975
fungi-Ascomycota.XP_003871375
fungi-Ascomycota.XP_002770816
fungi-Ascomycota.XP_001386755
fungi-Ascomycota.XP_006686571
fungi-Ascomycota.XP_020075015
fungi-Ascomycota.XP_018988034
fungi-Ascomycota.XP_001487569
fungi-Ascomycota.XP_019039754
fungi-Ascomycota.XP_011277169
fungi-Ascomycota.XP_020069053
fungi-Ascomycota.XP_003956458
fungi-Ascomycota.XP_018736903
fungi-Ascomycota.XP_013020355
fungi-Ascomycota.NP_588044
fungi-Ascomycota.XP_002838983
fungi-Basidiomycota.XP_007382434
fungi-Basidiomycota.XP_003036711
fungi-Basidiomycota.XP_002473355
fungi-Basidiomycota.XP_007392367
fungi-Basidiomycota.XP_007302991
fungi-Basidiomycota.XP_006460051
fungi-Basidiomycota.XP_007329042
Chlamydomonas_reinhardtii.XP_001700672
Volvox_carteri.XP_002947056
Monoraphidium_neglectum.XP_013906426
Chlamydomonas_eustigma.GAX78513
fungi-Chytridiomycota.XP_016609244
R. irregularis 40173
fungi-Mucoromycotina.XP_018284695
fungi-Microsporidia.XP_013237963
fungi-Microsporidia.XP_002995535
archaea-Thermococci.WP_068320532
archaea-Thermococci.WP_014733753
archaea-Thermococci.WP_013748534
archaea-Thermococci.WP_010868714
bac-Firmicutes.WP_036936801
bac-Firmicutes.WP_038287089
bac-Firmicutes.WP_013121724
bac-Firmicutes.WP_019225000
bac-Firmicutes.WP_015044926
bac-Firmicutes.WP_020491986
bac-Firmicutes.WP_034600830
Amoebozoa.XP_004259922
0.6

Supplementary Figure 14 Molecular phylogeny of translation factors. Plant sequences were obtained from BLASTP output (E value cutoff: 1e-3) and keyword search result. Numbers beside branches represent bootstrap values from maximum likelihood and Bayesian results, respectively. Asterisks indicate values lower than 50%. Scale bars represent substitution numbers per amino-acid site. Plant and fungal sequences are colored in green and blue, respectively.

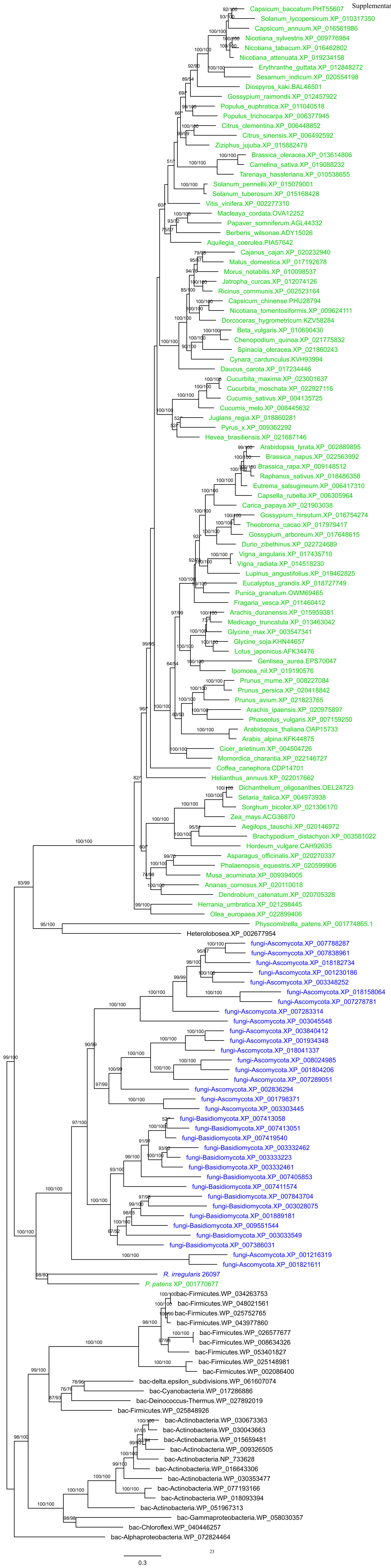
Capsicum_baccatum.PHT55607
Solanum_lycopersicum.XP_010317350
Capsicum_annuum.XP_016561986
Nicotiana_sylvestris.XP_009776984
Nicotiana_tabacum.XP_016462802
Nicotiana_attenuata.XP_019234158
Erythranthe_guttata.XP_012848272
Sesamum_indicum.XP_020554198
Diospyros_kaki.BAL46501
Gossypium_raimondii.XP_012457922
Populus_euphratica.XP_011040518
Populus_trichocarpa.XP_006377945
Citrus_clementina.XP_006448852
Citrus_sinensis.XP_006492592
Ziziphus_jujuba.XP_015882479
Brassica_oleracea.XP_013614806
Camelina_sativa.XP_019088232
Tarenaya_hassleriana.XP_010538655
Solanum_pennellii.XP_015079001
Solanum_tuberosum.XP_015168428
Vitis_vinifera.XP_002277310
Macleaya_cordata.OVA12252
Papaver_somniferum.AGL44332
Berberis_wilsonae.ADY15026
Aquilegia_coerulea.PIA57642
Cajanus_cajan.XP_020232940
Malus_domestica.XP_017192678
Morus_notabilis.XP_010098537
Jatropha_curcas.XP_012074126
Ricinus_communis.XP_002523164
Capsicum_chinense.PHU28794
Nicotiana_tomentosiformis.XP_009624111
Dorcoceras_hygrometricum.KZV58284
Beta_vulgaris.XP_010690430
Chenopodium_quinoa.XP_021775832
Spinacia_oleracea.XP_021860243
Cynara_cardunculus.KVH93994
Daucus_carota.XP_017234446
Cucurbita_maxima.XP_023001637
Cucurbita_moschata.XP_022927116
Cucumis_sativus.XP_004135725
Cucumis_melo.XP_008445632
Juglans_regia.XP_018860281
Pyrus_x.XP_009362292
Hevea_brasiliensis.XP_021687146
Arabidopsis_lyrata.XP_002889895
Brassica_napus.XP_022563992
Brassica_rapa.XP_009148512
Raphanus_sativus.XP_018486358
Eutrema_salsugineum.XP_006417310
Capsella_rubella.XP_006305964
Carica_papaya.XP_021903038
Gossypium_hirsutum.XP_016754274
Theobroma_cacao.XP_017979417
Gossypium_arboreum.XP_017648615
Durio_zibethinus.XP_022724689
Vigna_angularis.XP_017435710
Vigna_radiata.XP_014518230
Lupinus_angustifolius.XP_019462825
Eucalyptus_grandis.XP_018727749
Punica_granatum.OWM69465
Fragaria_vesca.XP_011460412
Arachis_duranensis.XP_015959381
Medicago_truncatula.XP_013463042
Glycine_max.XP_003547341
Glycine_soja.KHN44657
Lotus_japonicus.AFK34476
Genlisea_aurea.EPS70047
Ipomoea_nil.XP_019190576
Prunus_mume.XP_008227084
Prunus_persica.XP_020418842
Prunus_avium.XP_021823765
Arachis_ipaensis.XP_020975897
Phaseolus_vulgaris.XP_007159250
Arabidopsis_thaliana.OAP15733
Arabis_alpina.KFK44875
Cicer_arietinum.XP_004504726
Momordica_charantia.XP_022146727
Coffea_canephora.CDP14701
Helianthus_annuus.XP_022017662
Dichanthelium_oligosanthes.OEL24723
Setaria_italica.XP_004973938
Sorghum_bicolor.XP_021306170
Zea_mays.ACG36870
Aegilops_tauschii.XP_020146972
Brachypodium_distachyon.XP_003581022
Hordeum_vulgare.CAH92635
Asparagus_officinalis.XP_020270337
Phalaenopsis_equestris.XP_020599906
Musa_acuminata.XP_009394005
Ananas_comosus.XP_020110018
Dendrobium_catenatum.XP_020705328
Herrania_umbratica.XP_021298445
Olea_europaea.XP_022899406
Physcomitrella_patens.XP_001774865.1
Heterolobosea.XP_002677954
fungi-Ascomycota.XP_007788287
fungi-Ascomycota.XP_007838961
fungi-Ascomycota.XP_018182734
fungi-Ascomycota.XP_001230186
fungi-Ascomycota.XP_003348252
fungi-Ascomycota.XP_018158064
fungi-Ascomycota.XP_007278781
fungi-Ascomycota.XP_007283314
fungi-Ascomycota.XP_003045548
fungi-Ascomycota.XP_003840412
fungi-Ascomycota.XP_001934348
fungi-Ascomycota.XP_018041337
fungi-Ascomycota.XP_008024985
fungi-Ascomycota.XP_001804206
fungi-Ascomycota.XP_007289051
fungi-Ascomycota.XP_002836294
fungi-Ascomycota.XP_001798371
fungi-Ascomycota.XP_003303445
fungi-Basidiomycota.XP_007413058
fungi-Basidiomycota.XP_007413051
fungi-Basidiomycota.XP_007419540
fungi-Basidiomycota.XP_003332462
fungi-Basidiomycota.XP_003333223
fungi-Basidiomycota.XP_003332461
fungi-Basidiomycota.XP_007405853
fungi-Basidiomycota.XP_007411574
fungi-Basidiomycota.XP_007843704
fungi-Basidiomycota.XP_003028075
fungi-Basidiomycota.XP_001889181
fungi-Basidiomycota.XP_009551544
fungi-Basidiomycota.XP_003033549
fungi-Basidiomycota.XP_007386031
fungi-Ascomycota.XP_001216319
fungi-Ascomycota.XP_001821611
R. irregularis 26097
P. patens XP_001770677
bac-Firmicutes.WP_034263753
bac-Firmicutes.WP_048021561
bac-Firmicutes.WP_025752765
bac-Firmicutes.WP_043977860
bac-Firmicutes.WP_026577677
bac-Firmicutes.WP_008634326
bac-Firmicutes.WP_053401827
bac-Firmicutes.WP_025148981
bac-Firmicutes.WP_002086400
bac-delta.epsilon_subdivisions.WP_061607074
bac-Cyanobacteria.WP_017286886
bac-Deinococcus-Thermus.WP_027892019
bac-Firmicutes.WP_025848926
bac-Actinobacteria.WP_030673363
bac-Actinobacteria.WP_030043663
bac-Actinobacteria.WP_015659481
bac-Actinobacteria.WP_009326505
bac-Actinobacteria.NP_733628
bac-Actinobacteria.WP_016643306
bac-Actinobacteria.WP_030353477
bac-Actinobacteria.WP_077193166
bac-Actinobacteria.WP_018093394
bac-Actinobacteria.WP_051967313
bac-Gammaproteobacteria.WP_058030357
bac-Chloroflexi.WP_040446257
bac-Alphaproteobacteria.WP_072824464
0.3

Supplementary Figure 15 Molecular phylogeny of FAD-binding domain-containing proteins. Fungal sequences were obtained from BLASTP output (E value cutoff: 1e-3). Numbers beside branches represent bootstrap values from maximum likelihood and Bayesian results, respectively. Asterisks indicate values lower than 50%. Scale bars represent substitution numbers per amino-acid site. Plant and fungal sequences are colored in green and blue, respectively.

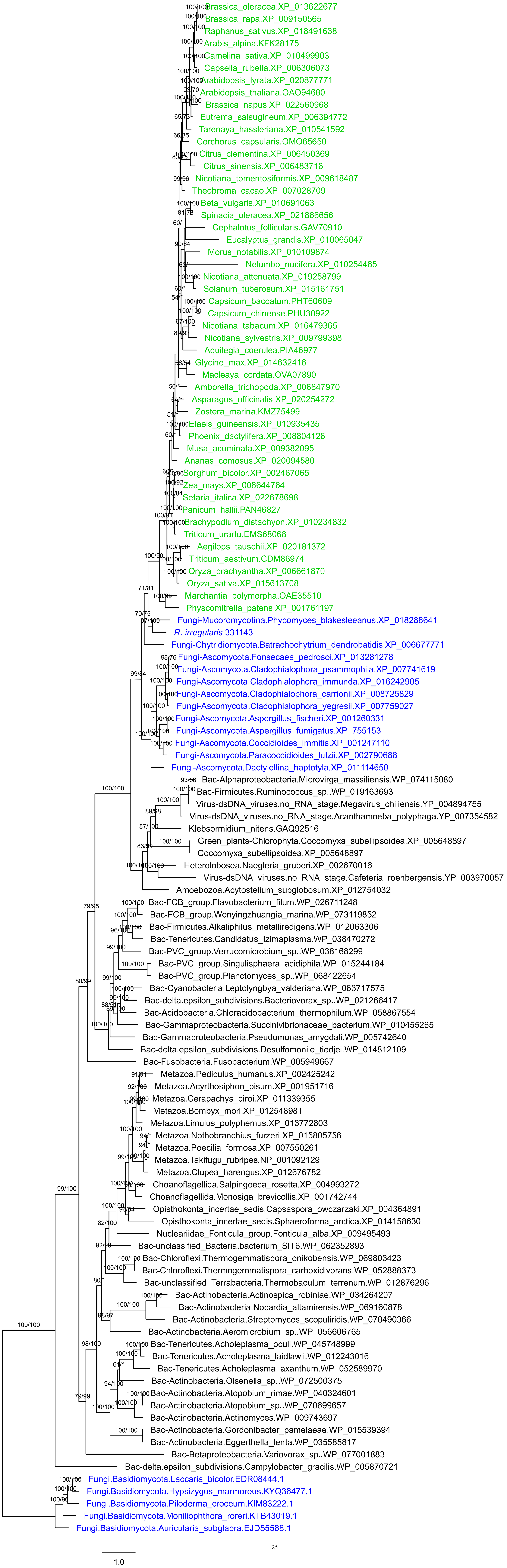
Brassica_oleracea.XP_013622677
Brassica_rapa.XP_009150565
Raphanus_sativus.XP_018491638
Arabis_alpina.KFK28175
Camelina_sativa.XP_010499903
Capsella_rubella.XP_006306073
Arabidopsis_lyrata.XP_020877771
Arabidopsis_thaliana.OAO94680
Brassica_napus.XP_022560968
Eutrema_salsugineum.XP_006394772
Tarenaya_hassleriana.XP_010541592
Corchorus_capsularis.OMO65650
Citrus_clementina.XP_006450369
Citrus_sinensis.XP_006483716
Nicotiana_tomentosiformis.XP_009618487
Theobroma_cacao.XP_007028709
Beta_vulgaris.XP_010691063
Spinacia_oleracea.XP_021866656
Cephalotus_follicularis.GAV70910
Eucalyptus_grandis.XP_010065047
Morus_notabilis.XP_010109874
Nelumbo_nucifera.XP_010254465
Nicotiana_attenuata.XP_019258799
Solanum_tuberosum.XP_015161751
Capsicum_baccatum.PHT60609
Capsicum_chinense.PHU30922
Nicotiana_tabacum.XP_016479365
Nicotiana_sylvestris.XP_009799398
Aquilegia_coerulea.PIA46977
Glycine_max.XP_014632416
Macleaya_cordata.OVA07890
Amborella_trichopoda.XP_006847970
Asparagus_officinalis.XP_020254272
Zostera_marina.KMZ75499
Elaeis_guineensis.XP_010935435
Phoenix_dactylifera.XP_008804126
Musa_acuminata.XP_009382095
Ananas_comosus.XP_020094580
Sorghum_bicolor.XP_002467065
Zea_mays.XP_008644764
Setaria_italica.XP_022678698
Panicum_hallii.PAN46827
Brachypodium_distachyon.XP_010234832
Triticum_urartu.EMS68068
Aegilops_tauschii.XP_020181372
Triticum_aestivum.CDM86974
Oryza_brachyantha.XP_006661870
Oryza_sativa.XP_015613708
Marchantia_polymorpha.OAE35510
Physcomitrella_patens.XP_001761197
Fungi-Mucoromycotina.Phycomyces_blakesleeanus.XP_018288641
R. irregularis 331143
Fungi-Chytridiomycota.Batrachochytrium_dendrobatidis.XP_006677771
Fungi-Ascomycota.Fonsecaea_pedrosoi.XP_013281278
Fungi-Ascomycota.Cladophialophora_psammophila.XP_007741619
Fungi-Ascomycota.Cladophialophora_immunda.XP_016242905
Fungi-Ascomycota.Cladophialophora_carrionii.XP_008725829
Fungi-Ascomycota.Cladophialophora_yegresii.XP_007759027
Fungi-Ascomycota.Aspergillus_fischeri.XP_001260331
Fungi-Ascomycota.Aspergillus_fumigatus.XP_755153
Fungi-Ascomycota.Coccidioides_immitis.XP_001247110
Fungi-Ascomycota.Paracoccidioides_lutzii.XP_002790688
Fungi-Ascomycota.Dactylellina_haptotyla.XP_011114650
Bac-Alphaproteobacteria.Microvirga_massiliensis.WP_074115080
Bac-Firmicutes.Ruminococcus_sp..WP_019163693
Virus-dsDNA_viruses.no_RNA_stage.Megavirus_chiliensis.YP_004894755
Virus-dsDNA_viruses.no_RNA_stage.Acanthamoeba_polyphaga.YP_007354582
Klebsormidium_nitens.GAQ92516
Green_plants-Chlorophyta.Coccomyxa_subellipsoidea.XP_005648897
Coccomyxa_subellipsoidea.XP_005648897
Heterolobosea.Naegleria_gruberi.XP_002670016
Virus-dsDNA_viruses.no_RNA_stage.Cafeteria_roenbergensis.YP_003970057
Amoebozoa.Acytostelium_subglobosum.XP_012754032
Bac-FCB_group.Flavobacterium_filum.WP_026711248
Bac-FCB_group.Wenyingzhuangia_marina.WP_073119852
Bac-Firmicutes.Alkaliphilus_metalliredigens.WP_012063306
Bac-Tenericutes.Candidatus_Izimaplasma.WP_038470272
Bac-PVC_group.Verrucomicrobium_sp..WP_038168299
Bac-PVC_group.Singulisphaera_acidiphila.WP_015244184
Bac-PVC_group.Planctomyces_sp..WP_068422654
Bac-Cyanobacteria.Leptolyngbya_valderiana.WP_063717575
Bac-delta.epsilon_subdivisions.Bacteriovorax_sp..WP_021266417
Bac-Acidobacteria.Chloracidobacterium_thermophilum.WP_058867554
Bac-Gammaproteobacteria.Succinivibrionaceae_bacterium.WP_010455265
Bac-Gammaproteobacteria.Pseudomonas_amygdali.WP_005742640
Bac-delta.epsilon_subdivisions.Desulfomonile_tiedjei.WP_014812109
Bac-Fusobacteria.Fusobacterium.WP_005949667
Metazoa.Pediculus_humanus.XP_002425242
Metazoa.Acyrthosiphon_pisum.XP_001951716
Metazoa.Cerapachys_biroi.XP_011339355
Metazoa.Bombyx_mori.XP_012548981
Metazoa.Limulus_polyphemus.XP_013772803
Metazoa.Nothobranchius_furzeri.XP_015805756
Metazoa.Poecilia_formosa.XP_007550261
Metazoa.Takifugu_rubripes.NP_001092129
Metazoa.Clupea_harengus.XP_012676782
Choanoflagellida.Salpingoeca_rosetta.XP_004993272
Choanoflagellida.Monosiga_brevicollis.XP_001742744
Opisthokonta_incertae_sedis.Capsaspora_owczarzaki.XP_004364891
Opisthokonta_incertae_sedis.Sphaeroforma_arctica.XP_014158630
Nucleariidae_Fonticula_group.Fonticula_alba.XP_009495493
Bac-unclassified_Bacteria.bacterium_SIT6.WP_062352893
Bac-Chloroflexi.Thermogemmatispora_onikobensis.WP_069803423
Bac-Chloroflexi.Thermogemmatispora_carboxidivorans.WP_052888373
Bac-unclassified_Terrabacteria.Thermobaculum_terrenum.WP_012876296
Bac-Actinobacteria.Actinospica_robiniae.WP_034264207
Bac-Actinobacteria.Nocardia_altamirensis.WP_069160878
Bac-Actinobacteria.Streptomyces_scopuliridis.WP_078490366
Bac-Actinobacteria.Aeromicrobium_sp..WP_056606765
Bac-Tenericutes.Acholeplasma_oculi.WP_045748999
Bac-Tenericutes.Acholeplasma_laidlawii.WP_012243016
Bac-Tenericutes.Acholeplasma_axanthum.WP_052589970
Bac-Actinobacteria.Olsenella_sp..WP_072500375
Bac-Actinobacteria.Atopobium_rimae.WP_040324601
Bac-Actinobacteria.Atopobium_sp..WP_070699657
Bac-Actinobacteria.Actinomyces.WP_009743697
Bac-Actinobacteria.Gordonibacter_pamelaeae.WP_015539394
Bac-Actinobacteria.Eggerthella_lenta.WP_035585817
Bac-Betaproteobacteria.Variovorax_sp..WP_077001883
Bac-delta.epsilon_subdivisions.Campylobacter_gracilis.WP_005870721
Fungi.Basidiomycota.Laccaria_bicolor.EDR08444.1
Fungi.Basidiomycota.Hypsizygus_marmoreus.KYQ36477.1
Fungi.Basidiomycota.Piloderma_croceum.KIM83222.1
Fungi.Basidiomycota.Moniliophthora_roreri.KTB43019.1
Fungi.Basidiomycota.Auricularia_subglabra.EJD55588.1
1.0

Supplementary Figure 16 Molecular phylogeny of flotillin-like proteins. Plant sequences were obtained from BLASTP output (E value cutoff: 1e-3). Fungal sequences were obtained from BLASTP output (E value cutoff: 1e-3) and keyword search result. Numbers beside branches represent bootstrap values from maximum likelihood and Bayesian results, respectively. Asterisks indicate values lower than 50%. Scale bars represent substitution numbers per amino-acid site. Plant and fungal sequences are colored in green and blue, respectively.

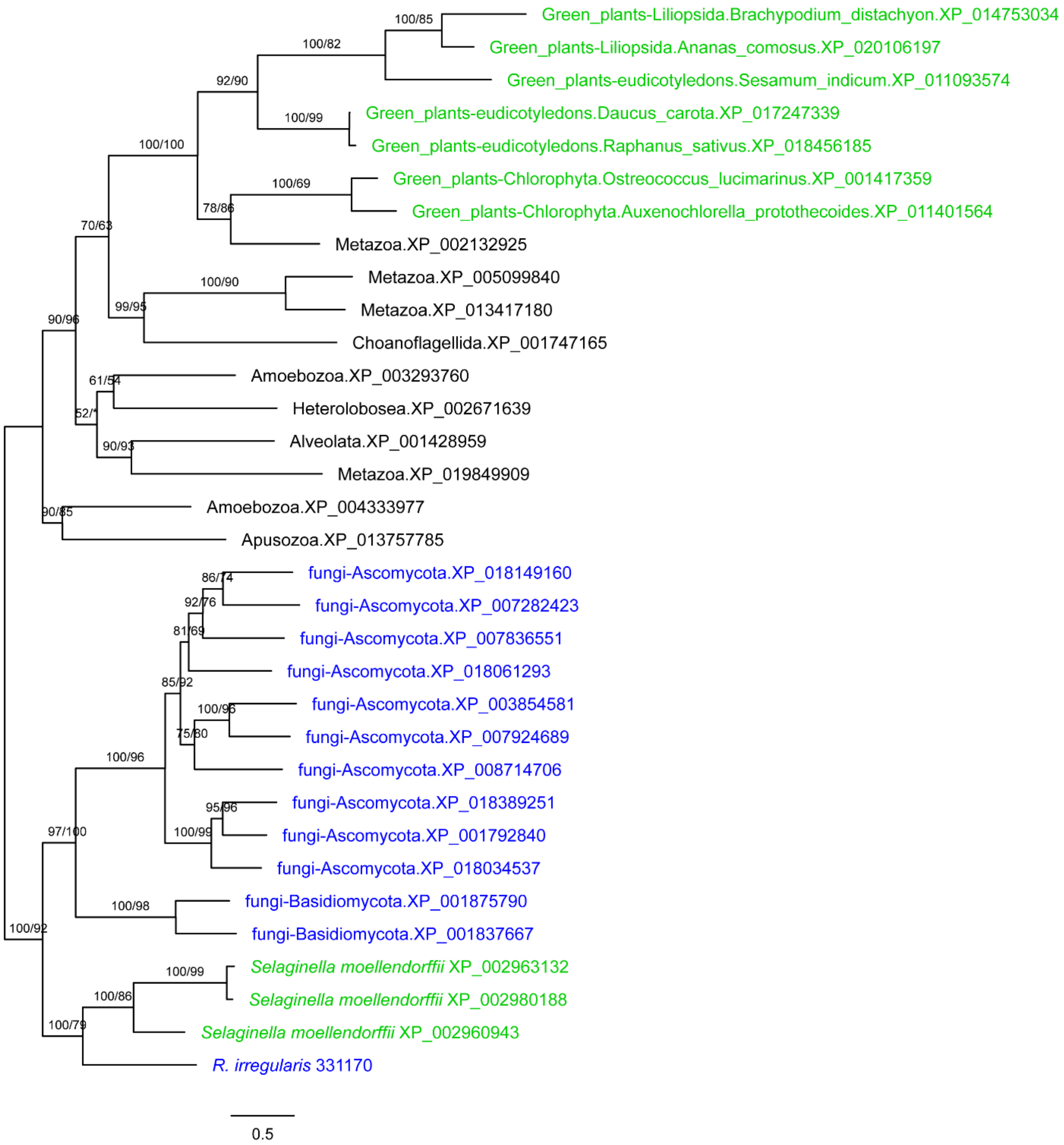

Supplementary Figure 17 Molecular phylogeny of ubiquitin-conjugating proteins. Plant sequences were obtained from BLASTP output (E value cutoff: 1e-3). Numbers beside branches represent bootstrap values from maximum likelihood and Bayesian results, respectively. Asterisks indicate

values lower than 50%. Scale bars represent substitution numbers per amino-acid site. Plant and fungal sequences are colored in green and blue, respectively.

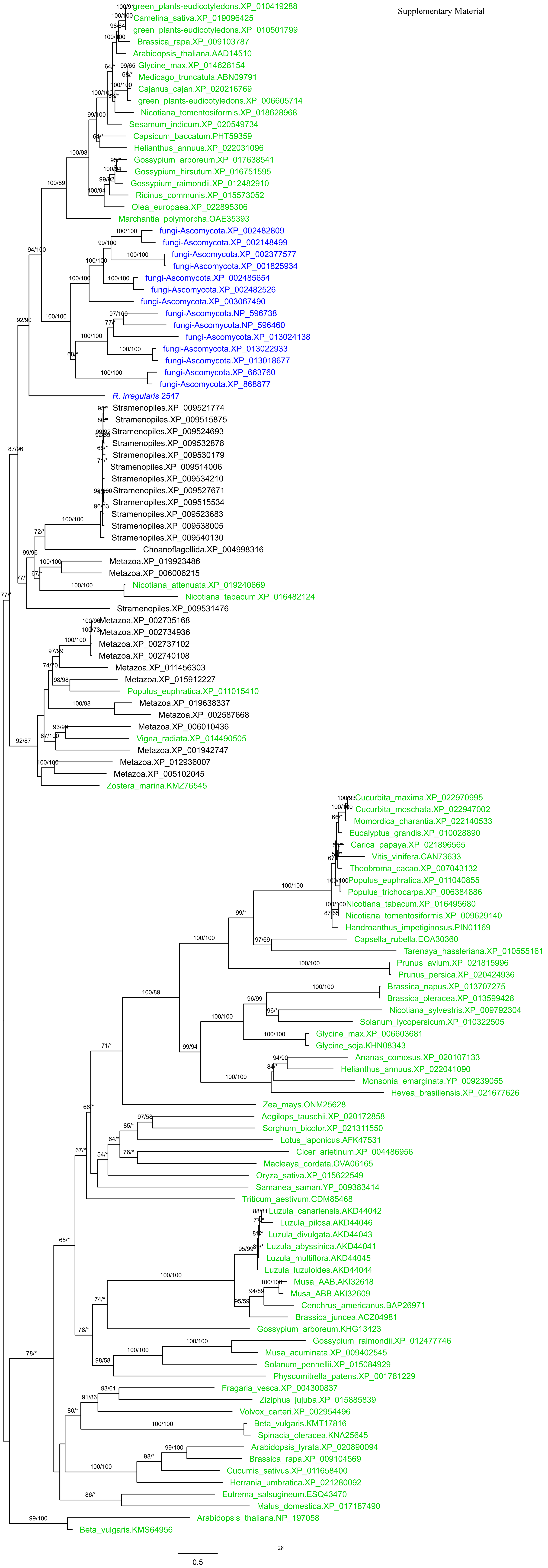
green_plants-eudicotyledons.XP_010419288
Camelina_sativa.XP_019096425
green_plants-eudicotyledons.XP_010501799
Brassica_rapa.XP_009103787
Arabidopsis_thaliana.AAD14510
Glycine_max.XP_014628154
Medicago_truncatula.ABN09791
Cajanus_cajan.XP_020216769
green_plants-eudicotyledons.XP_006605714
Nicotiana_tomentosiformis.XP_018628968
Sesamum_indicum.XP_020549734
Capsicum_baccatum.PHT59359
Helianthus_annuus.XP_022031096
Gossypium_arboreum.XP_017638541
Gossypium_hirsutum.XP_016751595
Gossypium_raimondii.XP_012482910
Ricinus_communis.XP_015573052
Olea_europaea.XP_022895306
Marchantia_polymorpha.OAE35393
fungi-Ascomycota.XP_002482809
fungi-Ascomycota.XP_002148499
fungi-Ascomycota.XP_002377577
fungi-Ascomycota.XP_001825934
fungi-Ascomycota.XP_002485654
fungi-Ascomycota.XP_002482526
fungi-Ascomycota.XP_003067490
fungi-Ascomycota.NP_596738
fungi-Ascomycota.NP_596460
fungi-Ascomycota.XP_013024138
fungi-Ascomycota.XP_013022933
fungi-Ascomycota.XP_013018677
fungi-Ascomycota.XP_663760
fungi-Ascomycota.XP_868877
R. irregularis 2547
Stramenopiles.XP_009521774
Stramenopiles.XP_009515875
Stramenopiles.XP_009524693
Stramenopiles.XP_009532878
Stramenopiles.XP_009530179
Stramenopiles.XP_009514006
Stramenopiles.XP_009534210
Stramenopiles.XP_009527671
Stramenopiles.XP_009515534
Stramenopiles.XP_009523683
Stramenopiles.XP_009538005
Stramenopiles.XP_009540130
Choanoflagellida.XP_004998316
Metazoa.XP_019923486
Metazoa.XP_006006215
Nicotiana_attenuata.XP_019240669
Nicotiana_tabacum.XP_016482124
Stramenopiles.XP_009531476
Metazoa.XP_002735168
Metazoa.XP_002734936
Metazoa.XP_002737102
Metazoa.XP_002740108
Metazoa.XP_011456303
Metazoa.XP_015912227
Populus_euphratica.XP_011015410
Metazoa.XP_019638337
Metazoa.XP_002587668
Metazoa.XP_006010436
Vigna_radiata.XP_014490505
Metazoa.XP_001942747
Metazoa.XP_012936007
Metazoa.XP_005102045
Zostera_marina.KMZ76545
Cucurbita_maxima.XP_022970995
Cucurbita_moschata.XP_022947002
Momordica_charantia.XP_022140533
Eucalyptus_grandis.XP_010028890
Carica_papaya.XP_021896565
Vitis_vinifera.CAN73633
Theobroma_cacao.XP_007043132
Populus_euphratica.XP_011040855
Populus_trichocarpa.XP_006384886
Nicotiana_tabacum.XP_016495680
Nicotiana_tomentosiformis.XP_009629140
Handroanthus_impetiginosus.PIN01169
Capsella_rubella.EOA30360
Tarenaya_hassleriana.XP_010555161
Prunus_avium.XP_021815996
Prunus_persica.XP_020424936
Brassica_napus.XP_013707275
Brassica_oleracea.XP_013599428
Nicotiana_sylvestris.XP_009792304
Solanum_lycopersicum.XP_010322505
Glycine_max.XP_006603681
Glycine_soja.KHN08343
Ananas_comosus.XP_020107133
Helianthus_annuus.XP_022041090
Monsonia_emarginata.YP_009239055
Hevea_brasiliensis.XP_021677626
Zea_mays.ONM25628
Aegilops_tauschii.XP_020172858
Sorghum_bicolor.XP_021311550
Lotus_japonicus.AFK47531
Cicer_arietinum.XP_004486956
Macleaya_cordata.OVA06165
Oryza_sativa.XP_015622549
Samanea_saman.YP_009383414
Triticum_aestivum.CDM85468
Luzula_canariensis.AKD44042
Luzula_pilosa.AKD44046
Luzula_divulgata.AKD44043
Luzula_abyssinica.AKD44041
Luzula_multiflora.AKD44045
Luzula_luzuloides.AKD44044
Musa_AAB.AKI32618
Musa_ABB.AKI32609
Cenchrus_americanus.BAP26971
Brassica_juncea.ACZ04981
Gossypium_arboreum.KHG13423
Gossypium_raimondii.XP_012477746
Musa_acuminata.XP_009402545
Solanum_pennellii.XP_015084929
Physcomitrella_patens.XP_001781229
Fragaria_vesca.XP_004300837
Ziziphus_jujuba.XP_015885839
Volvox_carteri.XP_002954496
Beta_vulgaris.KMT17816
Spinacia_oleracea.KNA25645
Arabidopsis_lyrata.XP_020890094
Brassica_rapa.XP_009104569
Cucumis_sativus.XP_011658400
Herrania_umbratica.XP_021280092
Eutrema_salsugineum.ESQ43470
Malus_domestica.XP_017187490
Arabidopsis_thaliana.NP_197058
Beta_vulgaris.KMS64956
0.5

Supplementary Figure 18 Molecular phylogeny of centromere protein B. Plant sequences were obtained from BLASTP output (E value cutoff: 1e-3) and keyword search result. Numbers beside branches represent bootstrap values from maximum likelihood and Bayesian results, respectively. Asterisks indicate values lower than 50%. Scale bars represent substitution numbers per amino-acid site. Plant and fungal sequences are colored in green and blue, respectively.

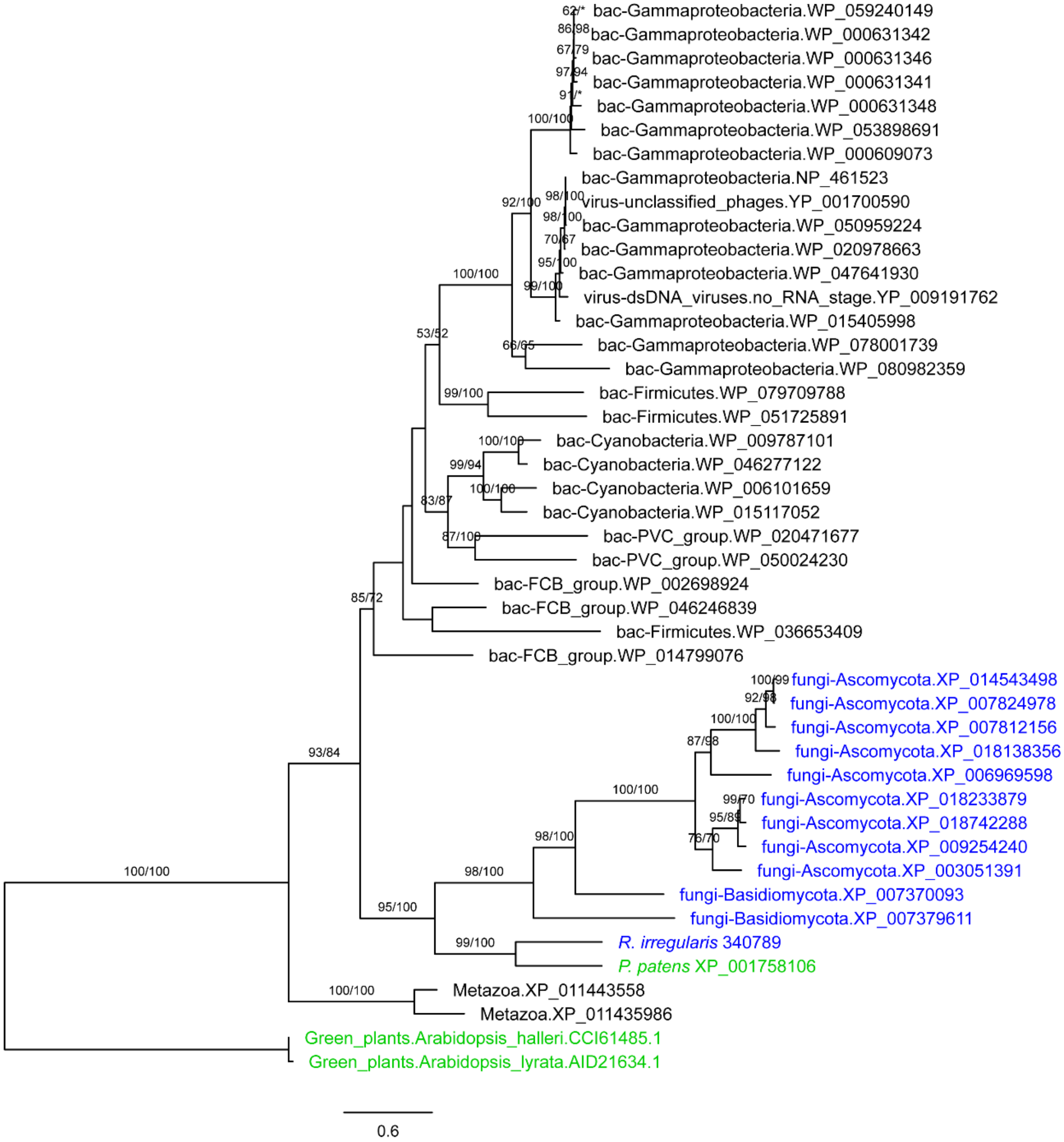

Supplementary Figure 19 Molecular phylogeny of macro domain-containing proteins. Plant sequences were obtained from BLASTP output (E value cutoff: 1). Numbers beside branches represent bootstrap values from maximum likelihood and Bayesian results, respectively. Asterisks indicate values lower than 50%. Scale bars represent substitution numbers per amino-acid site. Plant and fungal sequences are colored in green and blue, respectively.

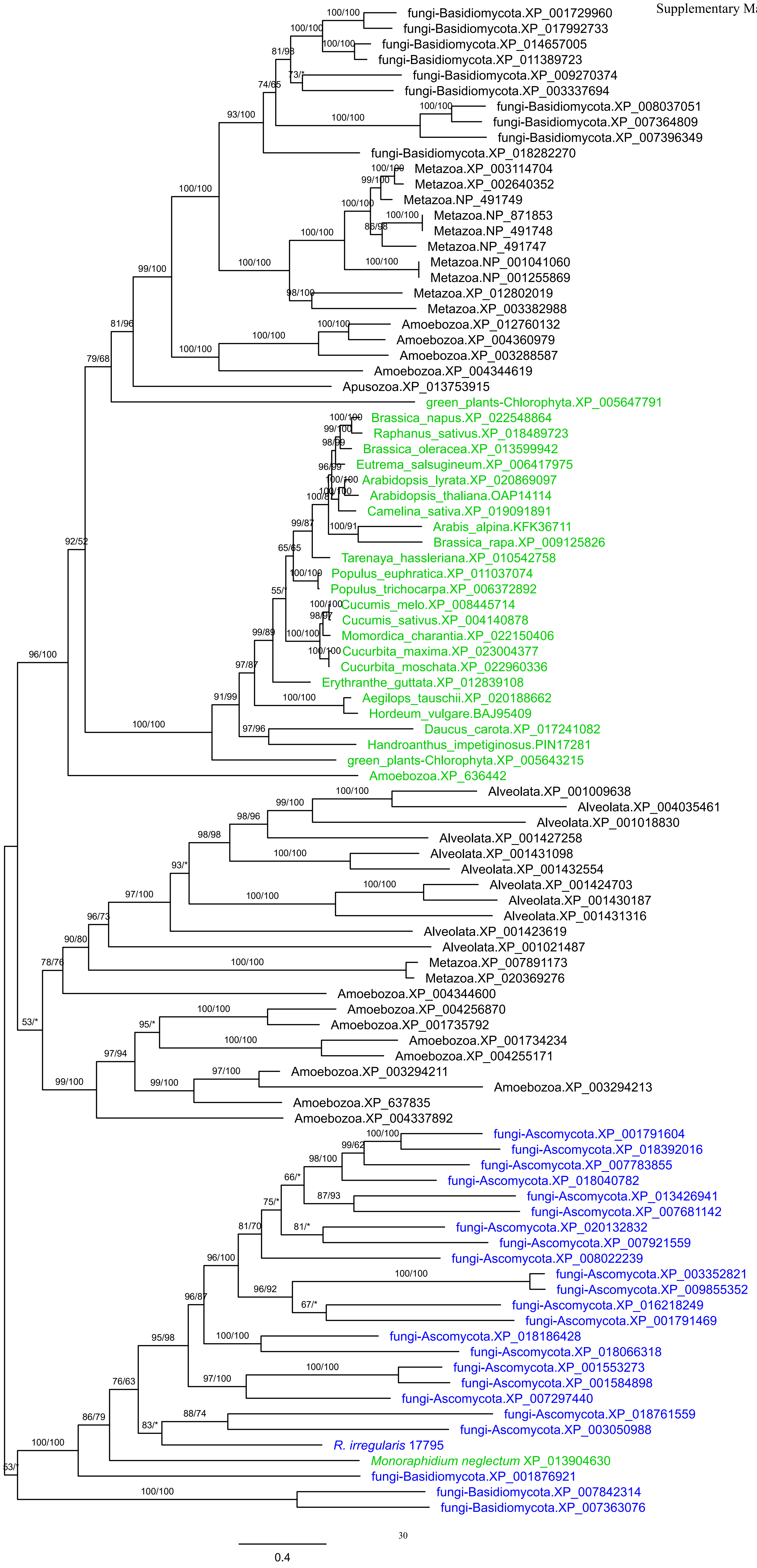
fungi-Basidiomycota.XP_001729960
fungi-Basidiomycota.XP_017992733
fungi-Basidiomycota.XP_014657005
fungi-Basidiomycota.XP_011389723
fungi-Basidiomycota.XP_009270374
fungi-Basidiomycota.XP_003337694
fungi-Basidiomycota.XP_008037051
fungi-Basidiomycota.XP_007364809
fungi-Basidiomycota.XP_007396349
fungi-Basidiomycota.XP_018282270
Metazoa.XP_003114704
Metazoa.XP_002640352
Metazoa.NP_491749
Metazoa.NP_871853
Metazoa.NP_491748
Metazoa.NP_491747
Metazoa.NP_001041060
Metazoa.NP_001255869
Metazoa.XP_012802019
Metazoa.XP_003382988
Amoebozoa.XP_012760132
Amoebozoa.XP_004360979
Amoebozoa.XP_003288587
Amoebozoa.XP_004344619
Apusozoa.XP_013753915
green_plants-Chlorophyta.XP_005647791
Brassica_napus.XP_022548864
Raphanus_sativus.XP_018489723
Brassica_oleracea.XP_013599942
Eutrema_salsugineum.XP_006417975
Arabidopsis_lyrata.XP_020869097
Arabidopsis_thaliana.OAP14114
Camelina_sativa.XP_019091891
Arabis_alpina.KFK36711
Brassica_rapa.XP_009125826
Tarenaya_hassleriana.XP_010542758
Populus_euphratica.XP_011037074
Populus_trichocarpa.XP_006372892
Cucumis_melo.XP_008445714
Cucumis_sativus.XP_004140878
Momordica_charantia.XP_022150406
Cucurbita_maxima.XP_023004377
Cucurbita_moschata.XP_022960336
Erythranthe_guttata.XP_012839108
Aegilops_tauschii.XP_020188662
Hordeum_vulgare.BAJ95409
Daucus_carota.XP_017241082
Handroanthus_impetiginosus.PIN17281
green_plants-Chlorophyta.XP_005643215
Amoebozoa.XP_636442
Alveolata.XP_001009638
Alveolata.XP_004035461
Alveolata.XP_001018830
Alveolata.XP_001427258
Alveolata.XP_001431098
Alveolata.XP_001432554
Alveolata.XP_001424703
Alveolata.XP_001430187
Alveolata.XP_001431316
Alveolata.XP_001423619
Alveolata.XP_001021487
Metazoa.XP_007891173
Metazoa.XP_020369276
Amoebozoa.XP_004344600
Amoebozoa.XP_004256870
Amoebozoa.XP_001735792
Amoebozoa.XP_001734234
Amoebozoa.XP_004255171
Amoebozoa.XP_003294211
Amoebozoa.XP_003294213
Amoebozoa.XP_637835
Amoebozoa.XP_004337892
fungi-Ascomycota.XP_001791604
fungi-Ascomycota.XP_018392016
fungi-Ascomycota.XP_007783855
fungi-Ascomycota.XP_018040782
fungi-Ascomycota.XP_013426941
fungi-Ascomycota.XP_007681142
fungi-Ascomycota.XP_020132832
fungi-Ascomycota.XP_007921559
fungi-Ascomycota.XP_008022239
fungi-Ascomycota.XP_003352821
fungi-Ascomycota.XP_009855352
fungi-Ascomycota.XP_016218249
fungi-Ascomycota.XP_001791469
fungi-Ascomycota.XP_018186428
fungi-Ascomycota.XP_018066318
fungi-Ascomycota.XP_001553273
fungi-Ascomycota.XP_001584898
fungi-Ascomycota.XP_007297440
fungi-Ascomycota.XP_018761559
fungi-Ascomycota.XP_003050988
R. irregularis 17795
Monoraphidium neglectum XP_013904630
fungi-Basidiomycota.XP_001876921
fungi-Basidiomycota.XP_007842314
fungi-Basidiomycota.XP_007363076
0.4

Supplementary Figure 20 Molecular phylogeny of E3 ubiquitin-protein ligases. Plant sequences were obtained from BLASTP output (E value cutoff: 1). Numbers beside branches represent bootstrap values from maximum likelihood and Bayesian results, respectively. Asterisks indicate values lower than 50%. Scale bars represent substitution numbers per amino-acid site. Plant and fungal sequences are colored in green and blue, respectively.

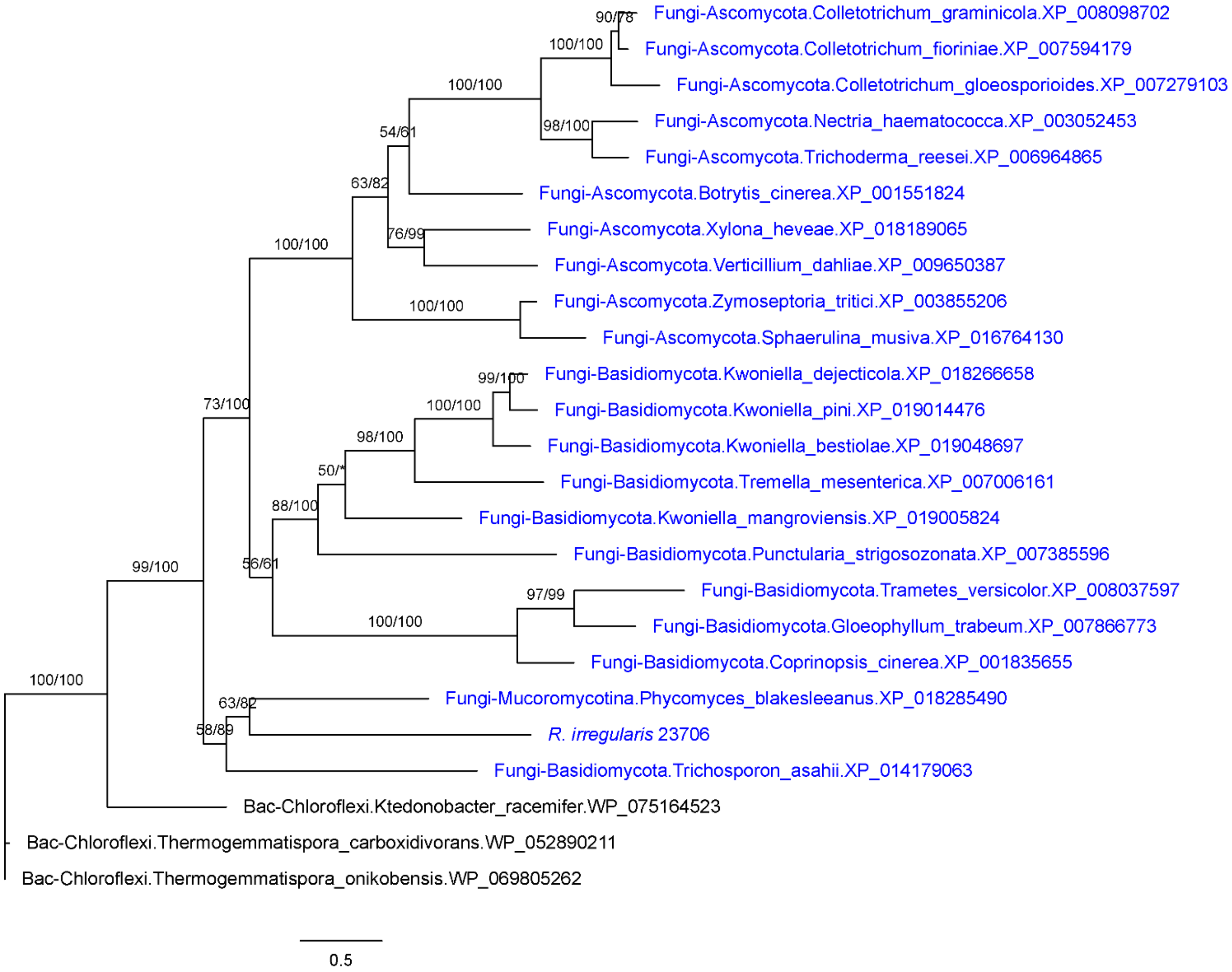

Supplementary Figure 21 Molecular phylogeny of methyltransferases. Numbers beside branches represent bootstrap values from maximum likelihood and Bayesian results, respectively. Asterisks indicate values lower than 50%. Scale bars represent substitution numbers per amino-acid site. Fungal sequences are colored in blue.

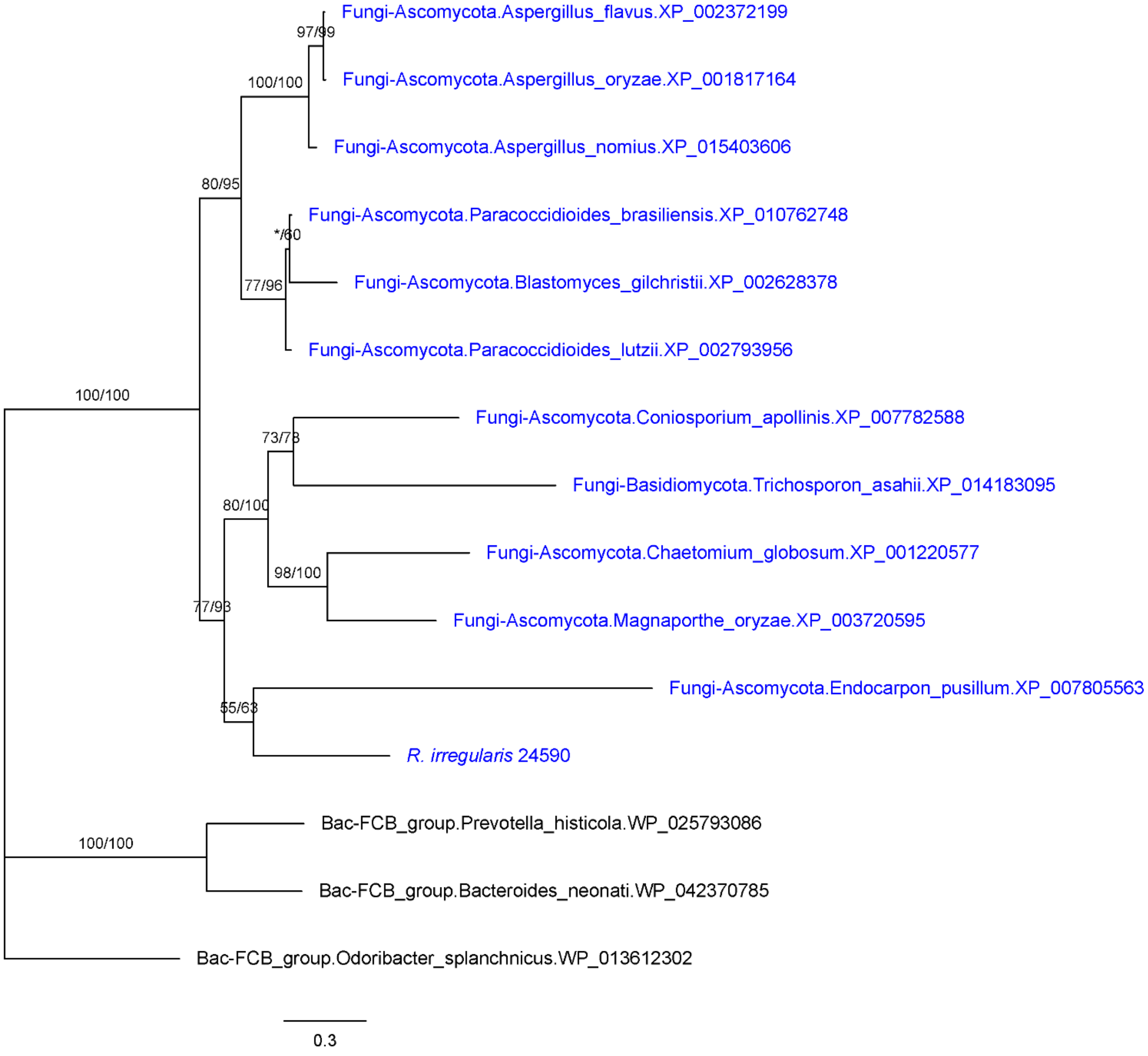

Supplementary Figure 22 Molecular phylogeny of unknown function protein ESA14777.1 (24590). Numbers beside branches represent bootstrap values from maximum likelihood and Bayesian results, respectively. Asterisks indicate values lower than 50%. Scale bars represent substitution numbers per amino-acid site. Fungal sequences are colored in blue.

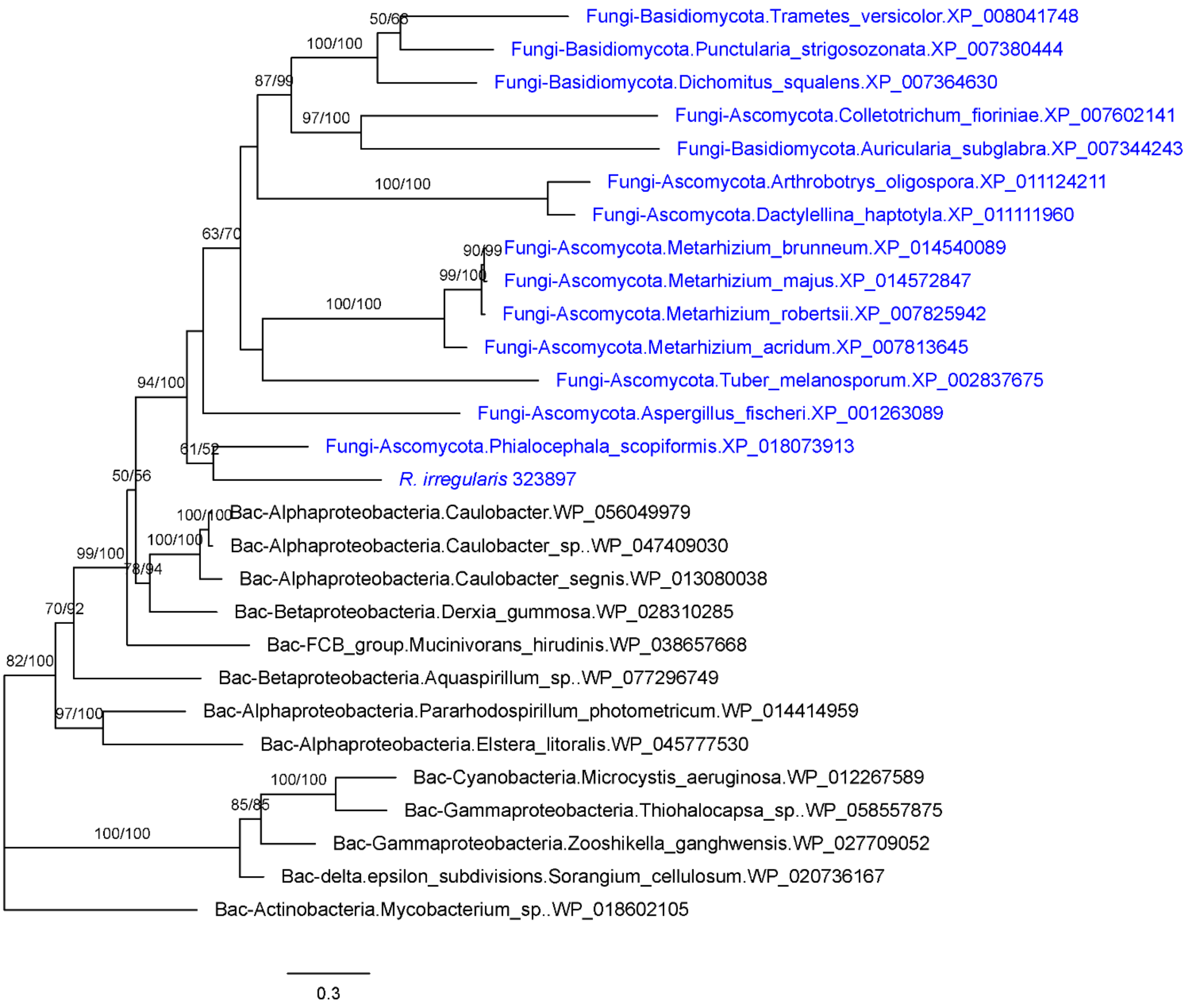

Supplementary Figure 23 Molecular phylogeny of phosphatidylserine decarboxylases. Numbers beside branches represent bootstrap values from maximum likelihood and Bayesian results, respectively. Asterisks indicate values lower than 50%. Scale bars represent substitution numbers per amino-acid site. Fungal sequences are colored in blue.

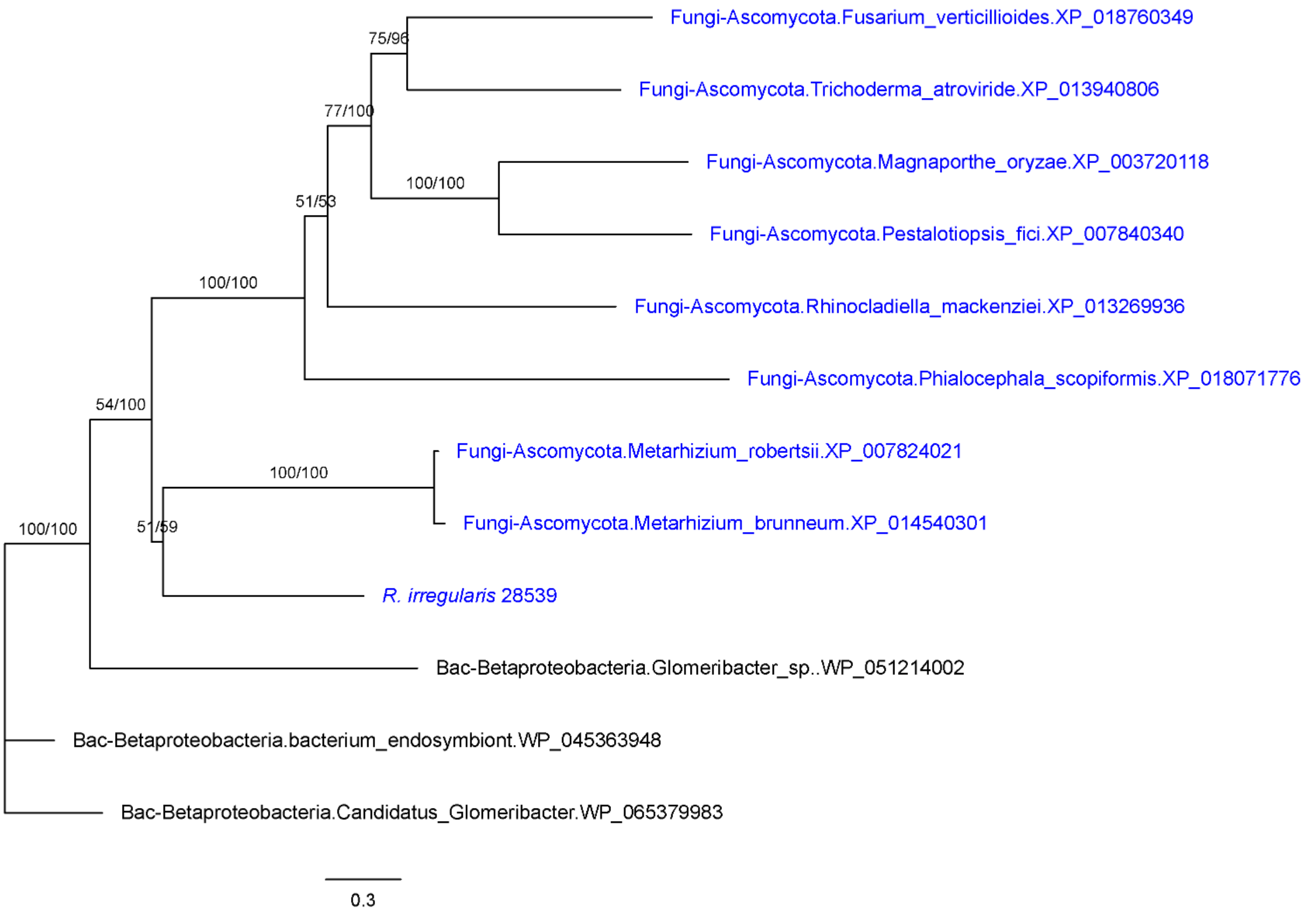

Supplementary Figure 24 Molecular phylogeny of nacht nucleoside triphosphatases. Numbers beside branches represent bootstrap values from maximum likelihood and Bayesian results, respectively. Asterisks indicate values lower than 50%. Scale bars represent substitution numbers per amino-acid site. Fungal sequences are colored in blue.

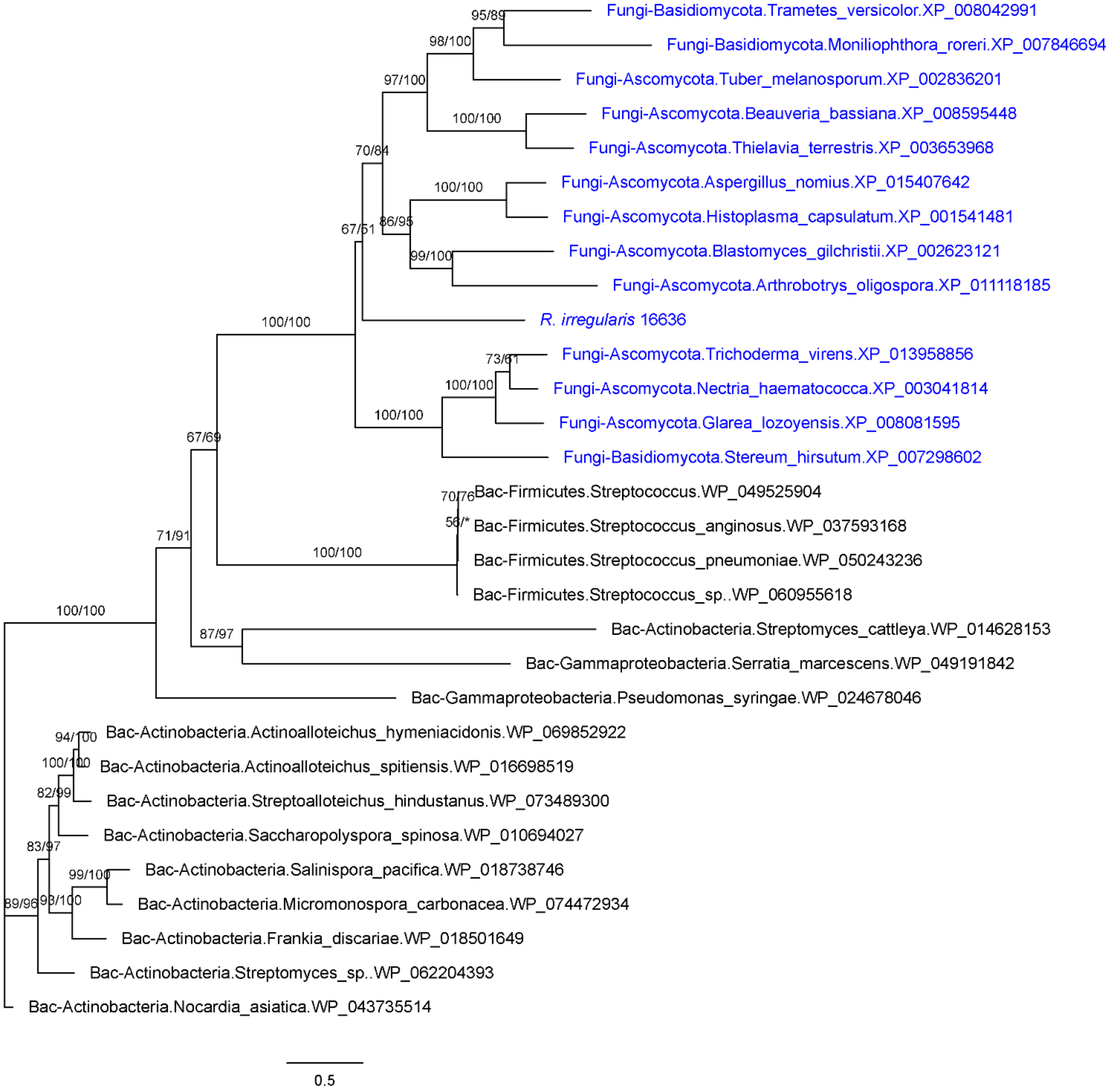

Supplementary Figure 25 Molecular phylogeny of unknown function protein ESA22231.1 (16636). Numbers beside branches represent bootstrap values from maximum likelihood and Bayesian results, respectively. Asterisks indicate values lower than 50%. Scale bars represent substitution numbers per amino-acid site. Fungal sequences are colored in blue.

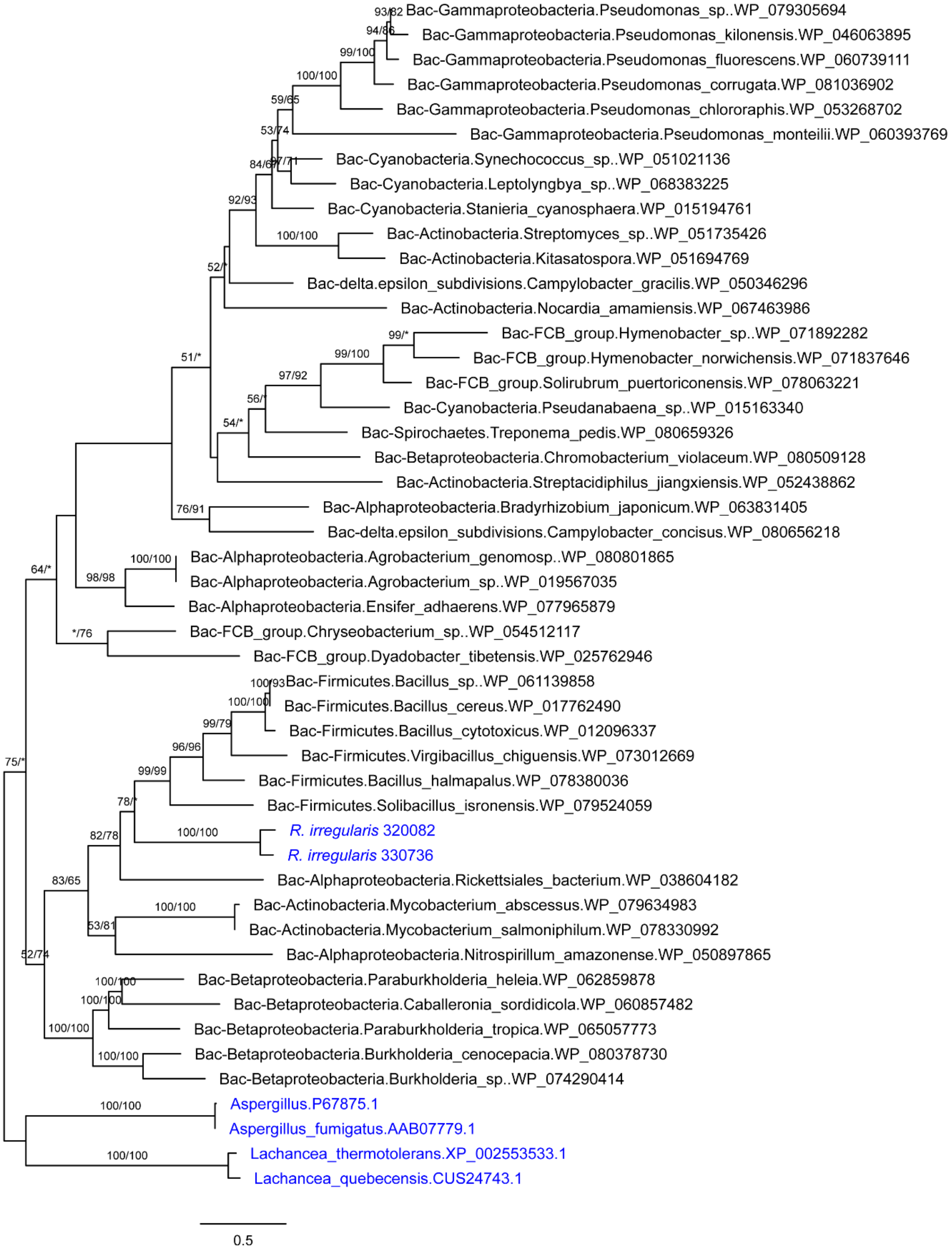

Supplementary Figure 26 Molecular phylogeny of cytotoxins. Fungal sequences were obtained from BLASTP output (E value cutoff: 1) and keyword search result. Numbers beside branches represent bootstrap values from maximum likelihood and Bayesian results, respectively. Asterisks indicate values lower than 50%. Scale bars represent substitution numbers per amino-acid site. Fungal sequences are colored in blue.
